# High‐Efficiency CO_2_ Electrolysis Enabled by Interface‐Engineered Composite Electrolytes in Ni‐Based SOEC

**DOI:** 10.1002/advs.202518091

**Published:** 2025-12-08

**Authors:** Rustam Yuldashev, Hyunchul Jung, Ji Hoon Park, Jin Hee Lee, Min‐Chul Kim

**Affiliations:** ^1^ CO_2_ & Energy Research Center Korea Research Institute of Chemical Technology (KRICT) Daejeon 34113 South Korea; ^2^ Advanced Materials and Chemical Engineering University of Science and Technology (UST) Daejeon 34114 South Korea

**Keywords:** CO_2_ electrolysis, delamination suppression, dip‐coating, solid oxide electrolyte cells, YSZ‐GDC composite layer

## Abstract

Interfacial instability between different electrolyte materials is a critical challenge hindering the commercialization of CO_2_ electrolysis via solid oxide electrolysis cells (SOECs). Specifically, the thermal deformation disparity at the yttria‐stabilized zirconia (YSZ) and Gd‐doped ceria (GDC) electrolyte interface leads to delamination during high‐temperature operation, severely degrading cell performance and durability. In this study, this issue is resolved by designing a novel composite intermediate layer, fabricated through a simple dip‐coating process using a mixture of YSZ and GDC powders. This composite layer effectively mitigates the thermal deformation disparity, ensuring excellent structural stability without delamination even after high‐temperature sintering. Consequently, the cell incorporating the composite interlayer exhibits a significantly reduced interfacial resistance and achieves an exceptional current density of 2.14 A cm^−2^ at 800 °C, which is among the highest performance levels reported for Ni‐based fuel electrode‐supported SOECs. Furthermore, the cell demonstrates excellent long‐term stability, maintaining 91% of its initial performance after 80 h of continuous operation under a harsh 1.6 V condition. The electrolyte layer also retains robust and stable interfacial adhesion, confirming the durability of the engineered interface. This study presents an effective electrolyte interface engineering strategy for the development of high‐performance and large‐area SOECs for CO_2_ electrolysis.

## Graphical abstract

This study addresses the critical issue of delamination between YSZ and GDC electrolytes in SOECs, which is caused by Thermal deformation disparity. A novel YSZ‐GDC composite interlayer, fabricated using a simple dip‐coating method, is introduced to solve this problem. This layer effectively suppresses delamination by mitigating thermal stress. The resulting cell achieves an exceptional current density of 2.14 A cm^−2^ and maintains 91% of its performance after 80 h, demonstrating excellent stability.

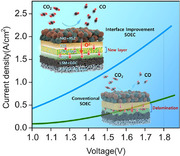

## 1 Introduction

**Scheme 1:**
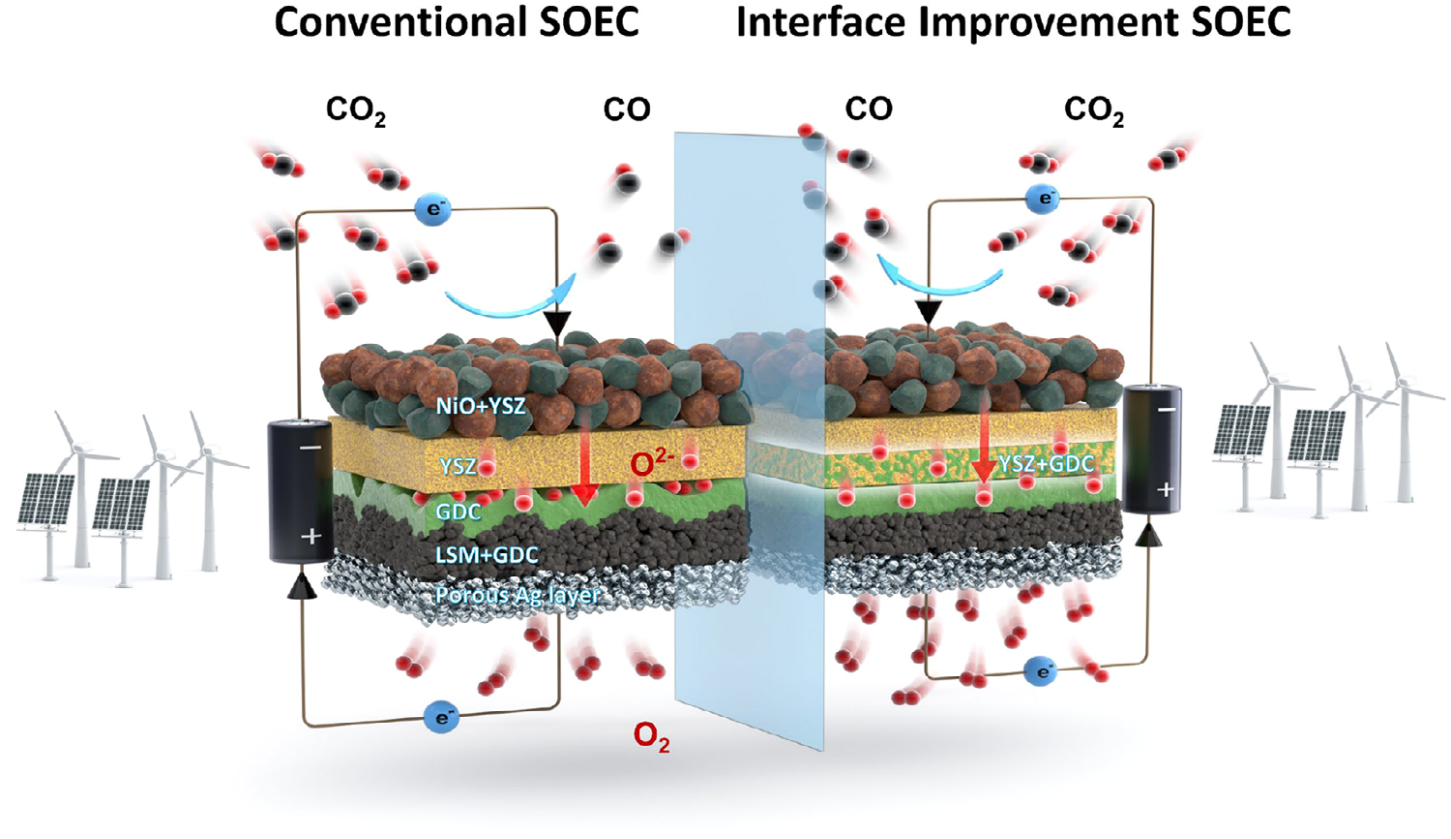
Schematic illustration of improved interfacial delamination and enhanced CO_2_ electrolysis performance achieved by a composite electrolyte layer.

The persistent global challenge of CO_2_ emissions is driving the more widespread adoption of carbon capture, utilization, and storage (CCUS) technologies.^[^ ^1^, ^2^, ^3^ ^]^ In this context, high‐temperature solid oxide electrolysis cells (HT‐SOECs) are recognized as a key technology within the CCUS framework, offering an efficient method to convert CO_2_ into chemical feedstocks powered by excess renewable electricity.^[^ ^4^, ^5^, ^6^ ^]^ HT‐SOEC is composed of an oxygen‐ion conductive electrolyte positioned between the fuel and oxygen electrodes, a configuration that demands precise coordination of the electrode‐electrolyte assembly.^[^ ^7^, ^8^, ^9^ ^]^ Enhancing SOEC performance through various approaches is essential for optimizing stack size, reducing costs, and lowering operating temperatures to mitigate cell degradation.^[^ ^10^, ^11^ ^]^ In SOECs, mixed ionic–electronic conductor (MIEC) electrodes such as lanthanum strontium manganite (LSM) and lanthanum strontium cobalt ferrite (LSCF) have been extensively investigated to mitigate electrode polarization.^[^ ^12^, ^13^ ^]^ However, when integrated with yttria‐stabilized zirconia (YSZ) electrolytes, insulating phases such as La_2_Zr_2_O_7_ and SrZrO_3_can form at the electrode–electrolyte interface.^[^ ^14^, ^15^ ^]^ To prevent these interfacial reactions, doped ceria materials such as Gd‐doped ceria (GDC) are employed as diffusion barrier layers, making YSZ/ceria bilayer structures essential for high‐performance SOECs.^[^ ^16^, ^17^, ^18^ ^]^ The significance of electrolyte barrier layers is particularly pronounced in large‐area Ni‐YSZ fuel electrode‐supported cells (FESC).^[^ ^19^, ^20^ ^]^ To successfully incorporate MIEC electrodes, stable ceria layers are necessary to mitigate interactions between MIEC and YSZ.^[^ ^21^, ^22^ ^]^ However, fabricating dense ceria‐based bilayers presents significant material and processing challenges, primarily due to delamination caused by thermal deformation disparity between YSZ (10.8 × 10^−^⁶ K^−1^) and GDC (14.1 × 10^−^⁶ K^−1^) during conventional high‐temperature sintering (≈1400 °C).^[^ ^23^, ^24^, ^25^, ^26^ ^]^ Such delamination substantially reduces interlayer adhesion, compromising the durability and performance of SOEC systems.^[^ ^27^, ^28^ ^]^ To address these challenges, various coating techniques, including dip coating, physical vapor deposition (PVD), pulsed laser deposition (PLD), and cold isostatic pressing (CIP), have been explored. While each method offers unique advantages, issues such as high equipment costs (PVD), complex processing parameters (PLD), and scalability limitations (CIP) persist.^[^ ^29^, ^30^, ^31^, ^32^ ^]^ In contrast, dip coating stands out due to its simplicity and cost‐efficiency, making it suitable for large‐area cell applications.^[^ ^33^, ^34^ ^]^ However, traditional dip‐coating methods have been limited by cracks and delamination when multiple electrolyte layers are deposited. In this study, we developed a YSZ‐GDC composite electrolyte coating solution specifically for dip coating, effectively addressing multi‐electrolyte delamination and improving adhesion (**Scheme** **1**). Notably, we directly dispersed YSZ and GDC powders into the solvent without high‐temperature pre‐sintering, optimizing the solution concentration to achieve continuous, multi‐layered electrolytes.

This approach effectively mitigated thermal deformation disparity between YSZ and GDC, overcoming interlayer delamination. Our results demonstrate that optimizing multi‐electrolyte synthesis via dip coating not only eliminates delamination but also significantly improves the CO_2_ electrolysis reaction. Enhancements in mechanical strength, adhesion, and ionic conductivity boosted oxygen ion mobility and overall SOEC efficiency. Furthermore, this work establishes a reliable platform for the subsequent performance optimization phase, offering a clear and practical pathway toward the development of high‐performance, large‐area Ni‐based SOECs.

## 2 Experimental Section

### 2.1 Fabrication of Solid Oxide Electrolysis Cell (SOEC)

The SOEC was fabricated using a NiO‐YSZ‐based fuel electrode‐supported structure. 8YSZ powder (8 mol% Y_2_O_3,_ Tosoh), NiO powder (JT Baker), graphite pore‐former (particle size < 20 µm, Sigma–Aldrich), and polyvinyl butyral (PVB, Sigma–Aldrich) binder were mixed in ethanol at a weight ratio of 0.48:0.4:0.095:0.025 and ball‐milled for 3 days for uniform dispersion. After milling, the mixture was dried at 90 °C in a vacuum oven to remove residual ethanol. A catalyst powder (1.9 g) was pressed into a cylindrical mold (diameter: 25 mm) at 5 tons for 2 min. The green pellets were then sequentially heated at 400, 600, and 900 °C for 2 h each. Subsequently, the pellets were dip‐coated in cathode functional layer (CFL) slurry, YSZ slurry, and YSZ‐GDC composite slurry, with each coating undergoing identical heat treatment (400, 600, and 900 °C for 2 h). The YSZ–GDC slurry was prepared by mixing YSZ and GDC powders with polyvinyl butyral (PVB) as a binder, dibutyl phthalate as a plasticizer (Sigma–Aldrich), Triton X‐100 as a homogenizer (Sigma–Aldrich), fish oil as a dispersant (Sigma–Aldrich), toluene (Sigma–Aldrich), and 2‐propanol (Sigma–Aldrich), followed by homogenization at 2000 rpm for 30 min (Figure S1a, Supporting Information). The slurry composition was adjusted according to the relative fractions (10‐x)YSZ:(x)GDC (x = 3, 5, 8, 9). Finally, pellets were dip‐coated in GDC slurry and pre‐sintered at 1380 °C for 3 h. The conventional SOEC anode composed of a strontium‐doped lanthanum manganite (LSM) and gadolinium‐doped ceria (GDC) composite was applied via screen printing and sintered at 1200 °C for 3 h. To further improve electronic conductivity, a porous silver (Ag) layer was finally deposited on the anode layer and subsequently sintered at 870 °C.^[^ ^35^ ^]^ The resulting SOEC structure was a NiO+YSZ (cathode) /NiO+YSZ(CFL)/YSZ/YSZ:GDC/GDC/LSM‐GDC(anode)/porous silver (Ag) layer, with a diameter of 19.6 mm and an active electrode area of 1 cm^2^. Figure S1b (Supporting Information) illustrates cell morphology at each synthesis stage.

### 2.2 Characterization Methods

The microstructure was characterized using scanning electron microscopy (SEM; JSM‐7610F Plus). Surface morphology, roughness, and height of electrolyte layers were analyzed using atomic force microscopy (AFM; Dimension Edge AFM, Bruker Corp.). Crystal structures of YSZ/GDC interfaces were analyzed at room temperature by X‐ray diffraction (XRD; D8 ADVANCE, Bruker Corp.). The dimensional change (expansion and shrinkage behavior) of YSZ, GDC, and their composite was measured using a dilatometer (DIL 402C, NETZSCH, Germany) from room temperature to 1500 °C. Surface chemical properties were investigated using X‐ray photoelectron spectroscopy (XPS; NEXSA‐G2, Thermo Scientific). Raman spectra were obtained using a confocal Raman microscope (LabRAM HR Evolution, Horiba Scientific). Electrochemical properties and electrode resistances were measured at temperatures ranging from 600 to 800 °C using a four‐probe potentiostat (SP‐300, Bio‐Logic) under a CO_2_ flow rate of 50 sccm, with a 100 mV AC amplitude signal and various applied voltages. Impedance spectroscopy measurements were performed from 0.1 kHz to 100 MHz, with distribution of relaxation times (DRT) analyses conducted using the DRT Tools software developed by Ciucci et al.^[^ ^36^ ^]^ Current‐voltage (*I–V*) characteristics of cells were measured using an in‐house single‐cell measurement system. Pt meshes (Alfa Aesar) and Ag paste (Elcoat) were used for current collection on both electrodes. Prior to testing, the cathode compartment was purged with N_2_, then switched to hydrogen to reduce NiO to Ni at 800 °C. During electrochemical testing, CO_2_ was supplied at 50 sccm to the fuel electrode, while the oxygen electrode was exposed to ambient air. CO_2_ and CO content in outlet gases were analyzed using an online gas chromatograph (iGC 7200, DS Science) equipped with a thermal conductivity detector (TCD). Gas flow rates were monitored using a flowmeter, and Faradaic efficiency (FE) was calculated using standard equations.^[^ ^37^ ^]^

The Faraday efficiency (FE) was calculated using the following formula:

(1)

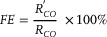

where $R_{CO}^{\prime }$ is the practical CO production rate as determined by GC analysis.

CO flow(ml/min) = 2.10652 × 10^−6^⋅(Peak area) Figure S2 (Supporting Information):

(2)

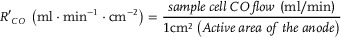




*R_CO_*, which represents the theoretical production of CO, is derived from the electrolytic current density using Equation (3):

(3) $$\begin{array}{cc} R_{CO} & =n\cdot \;V_{m}=\frac{i\;\cdot \;t}{F\;\cdot z}\;\cdot Vm\\ & =\frac{i\left(A\cdot cm^{-2}\right)\cdot t}{96465\;\left(C\cdot mol^{-1}\right)\cdot z}\;\cdot 22.4\;\times \;10^{3}\left(ml\cdot mol^{-1}\right)\\ & =\frac{i\left(A\cdot cm^{-2}\right)\cdot 60}{96465\;\left(s\cdot A\cdot mol^{-1}\right)\cdot z}\cdot 22.4\;\times \;10^{3}\left(ml\cdot mol^{-1}\right)\\ & =6.956\;\cdot i\left(ml\cdot min^{-1}\cdot cm^{-2}\right) \end{array}$$

where *n* is the mole of production of CO on the catalyst surface, *i* is the electrolyte current density, *t* is the CO_2_ reduction reaction time, F is the Faraday constant (96465 C mol^−1^), *z* is the electron transfer number, and *z* is 2 in the CO_2_ reduction reaction.

## 3 Results and Discussion

### 3.1 Suppression of Interfacial Delamination Using YSZ–GDC Composite Layers

**Figure 1:**
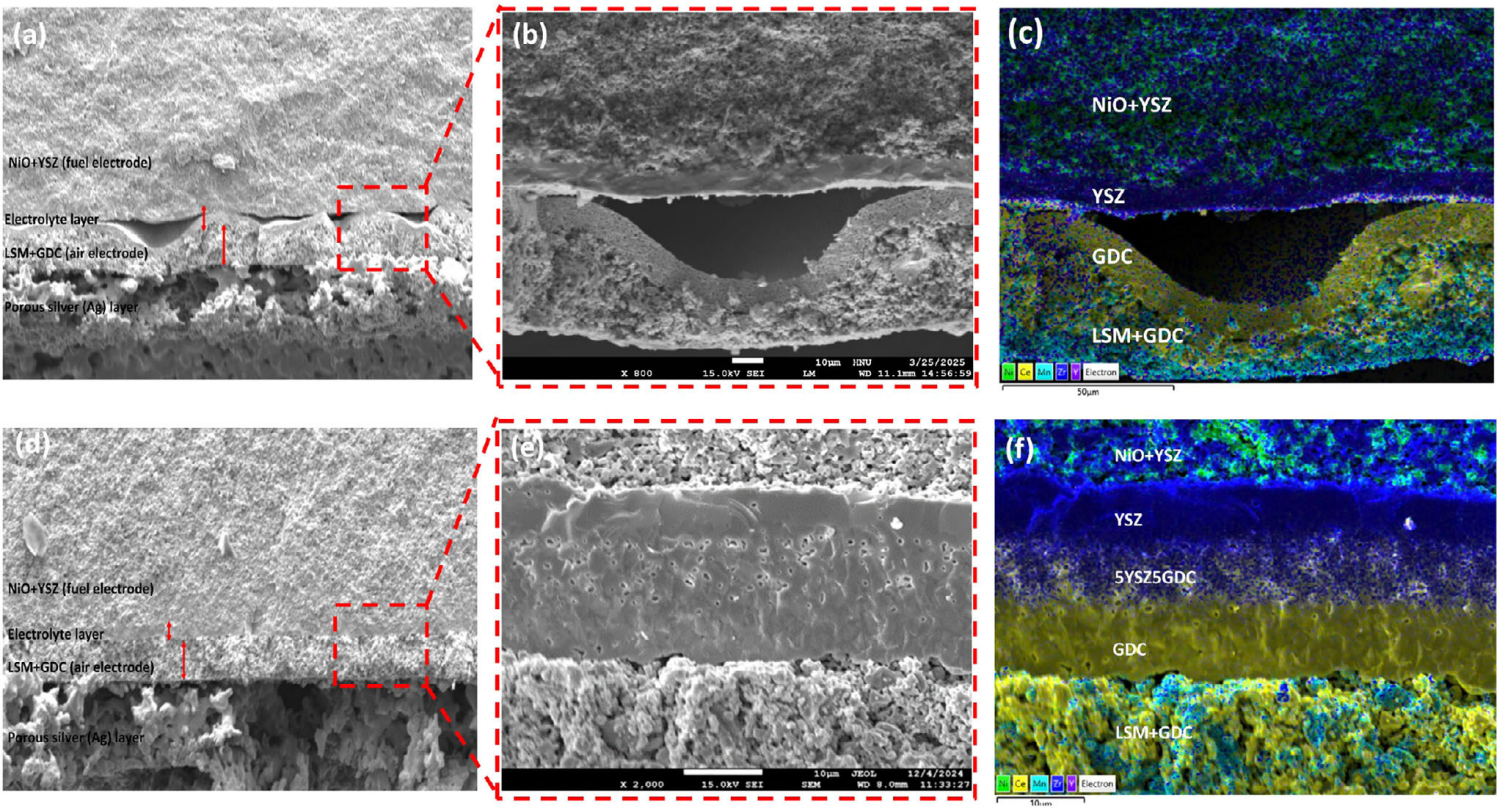
a,b) Cross‐sectional SEM images and c) EDS mapping of YSZ/GDC electrolyte bilayer after sintering at 1380 °C. d,e) Cross‐sectional SEM images and f) EDS mapping of YSZ/5YSZ5GDC/GDC composite electrolyte layer after sintering at 1380 °C.

**Figure 2:**
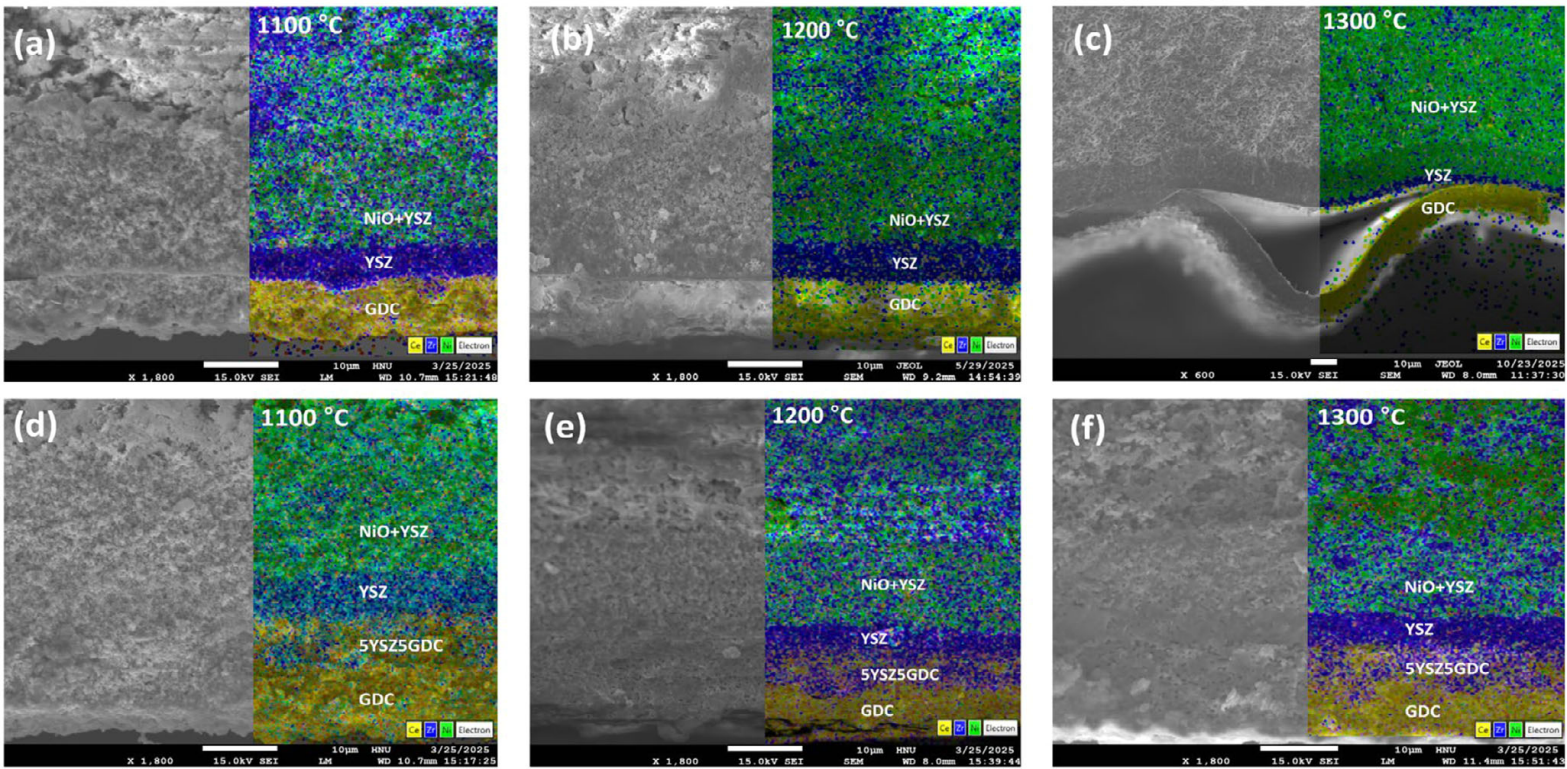
Cross‐sectional SEM images and EDS mappings of cells with YSZ/GDC electrolyte layers (a–c) and YSZ/5YSZ5GDC/GDC composite electrolyte layers (d–f) sintered at 1100, 1200, and 1300 °C.

**Figure 3:**
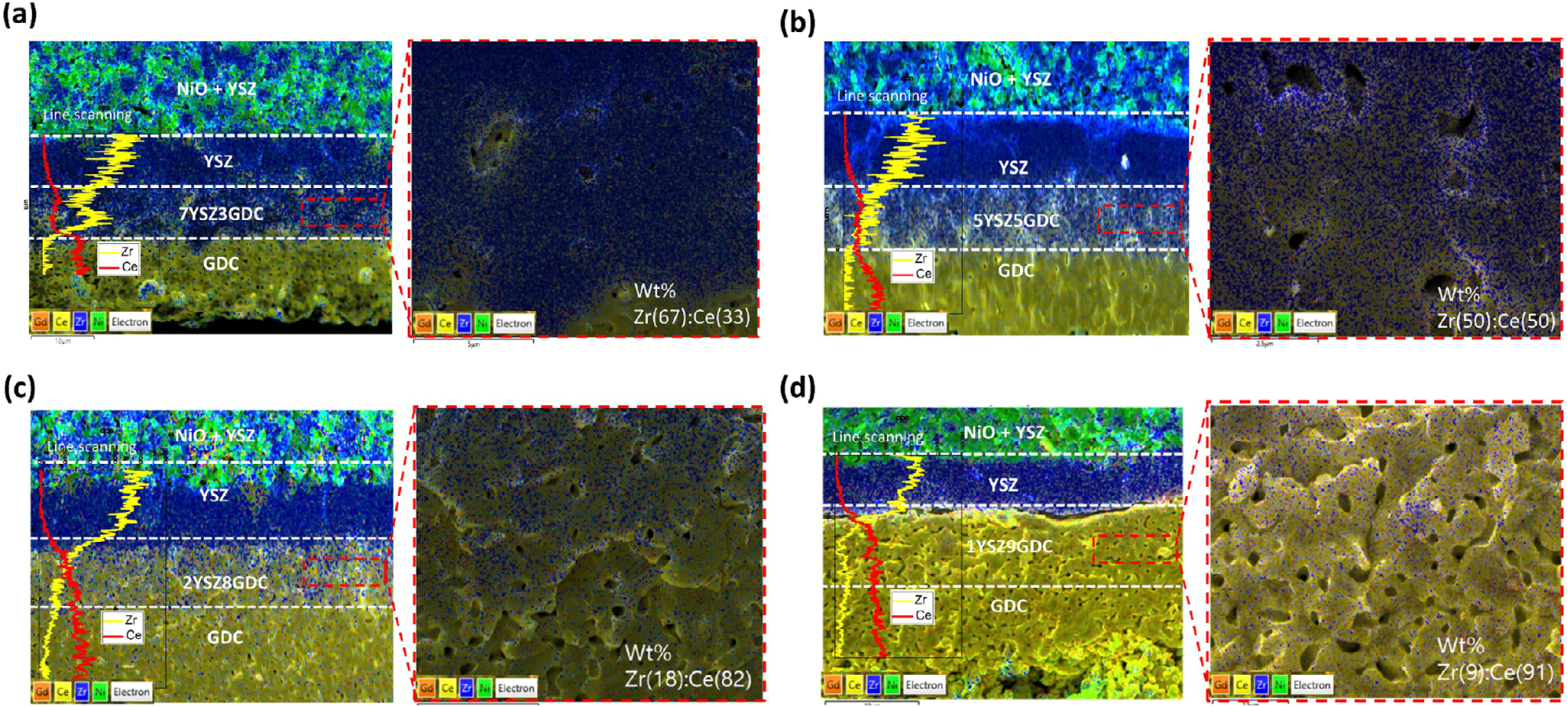
Cross‐sectional SEM images and corresponding EDS line scans (Zr, Ce) of cells with a) 7YSZ3GDC, b) 5YSZ5GDC, c) 2YSZ8GDC, and d) 1YSZ9GDC composite electrolyte layers.

In this study, a novel composite intermediate electrolyte layer consisting of yttria‐stabilized zirconia (YSZ) and gadolinium‐doped ceria (GDC) was designed for single‐cell configurations supported by YSZ and GDC‐based electrolytes. To achieve structural and material compatibility between YSZ and GDC electrolyte layers, composite intermediate layers (7YSZ3GDC, 5YSZ5GDC, 2YSZ8GDC, and 1YSZ9GDC) were fabricated via dip‐coating, optimizing their chemical composition and electrochemical performance. Figure S3 (Supporting Information) demonstrates stable adhesion without delamination in cells without the composite intermediate layer after sintering at 900 °C. However, significant delamination occurred at the higher sintering temperature of 1380 °C (**Figure** **1**a–c; Figure S4a, Supporting Information). This is attributed to the thermal deformation disparity between YSZ and GDC, which arises not only from different thermal expansion coefficients but also from incompatible sintering shrinkage behaviors.^[^ ^38^ ^]^ As shown by dilatometric analysis (Figure S5, Supporting Information), YSZ undergoes rapid densification starting near 1150 °C, while GDC begins gradual shrinkage ≈950 °C. This combined influence of thermal expansion and sintering incompatibility leads to significant thermomechanical stress and delamination at high co‐sintering temperatures. To overcome this issue, the composite electrolyte layer was developed and, notably, cells incorporating the 5YSZ5GDC composite remained well‐bonded without delamination even after sintering at 1380 °C (Figure 1d–f; Figure S4b, Supporting Information). To further investigate delamination phenomena and identify critical temperature ranges at which thermal deformation disparity was most significant, cells were fabricated and observed at various sintering temperatures (1100, 1200, 1300 °C). Cross‐sectional images (**Figure** **2**a–c) revealed that delamination in cells without the composite electrolyte began at 1300 °C and intensified with increased sintering temperature. Conversely, cells containing the 5YSZ5GDC composite maintained stable, undelaminated electrolyte layers across all tested temperatures (Figure 2d–f). These findings confirm that the composite intermediate layer effectively mitigates thermal deformation disparity between YSZ and GDC. As indicated by dilatometric analysis (Figure S5, Supporting Information), the 5YSZ5GDC composite itself exhibits an intermediate and smoother shrinkage curve compared to the distinct behaviors of pure YSZ and GDC. This demonstrates that the composite layer acts as a functional gradient, gradually synchronizing the densification kinetics of the two oxides and mitigating thermomechanical stress accumulation during co‐sintering, thus functioning as a critical interface that enhances the overall stability of the electrolyte assembly. To determine the optimal ratio of the composite intermediate layer composed of YSZ and GDC, we systematically varied their proportions and examined cross‐sectional and surface morphologies in detail (**Figure** **3**). Stable interfacial bonding between YSZ and the composite electrolyte layer was maintained up to the 2YSZ8GDC composition. However, further reduction in YSZ content to 1YSZ9GDC resulted in the formation of interfacial cracks, indicating weakened structural integrity (Figure 3d). To elucidate the role of YSZ content in maintaining interfacial integrity, the volume fractions of the components were calculated (see Table S1, Supporting Information). The 1YSZ9GDC composition contains only 11.7 vol% YSZ, which is below the typical percolation threshold of 20–30 vol% reported for ceramic composites.^[^ ^39^ ^]^

Below this threshold, YSZ cannot form a continuous structural network, resulting in isolated domains that fail to accommodate the thermal deformation disparity with the YSZ substrate.^[^ ^40^, ^41^ ^]^ This lack of percolation is the direct cause of the observed interfacial cracking (Figure 3d), which in turn compromises the cell's electrochemical performance.

### 3.2 Microstructural Stabilization of GDC Electrolytes via Composite Interlayers

**Figure 4:**
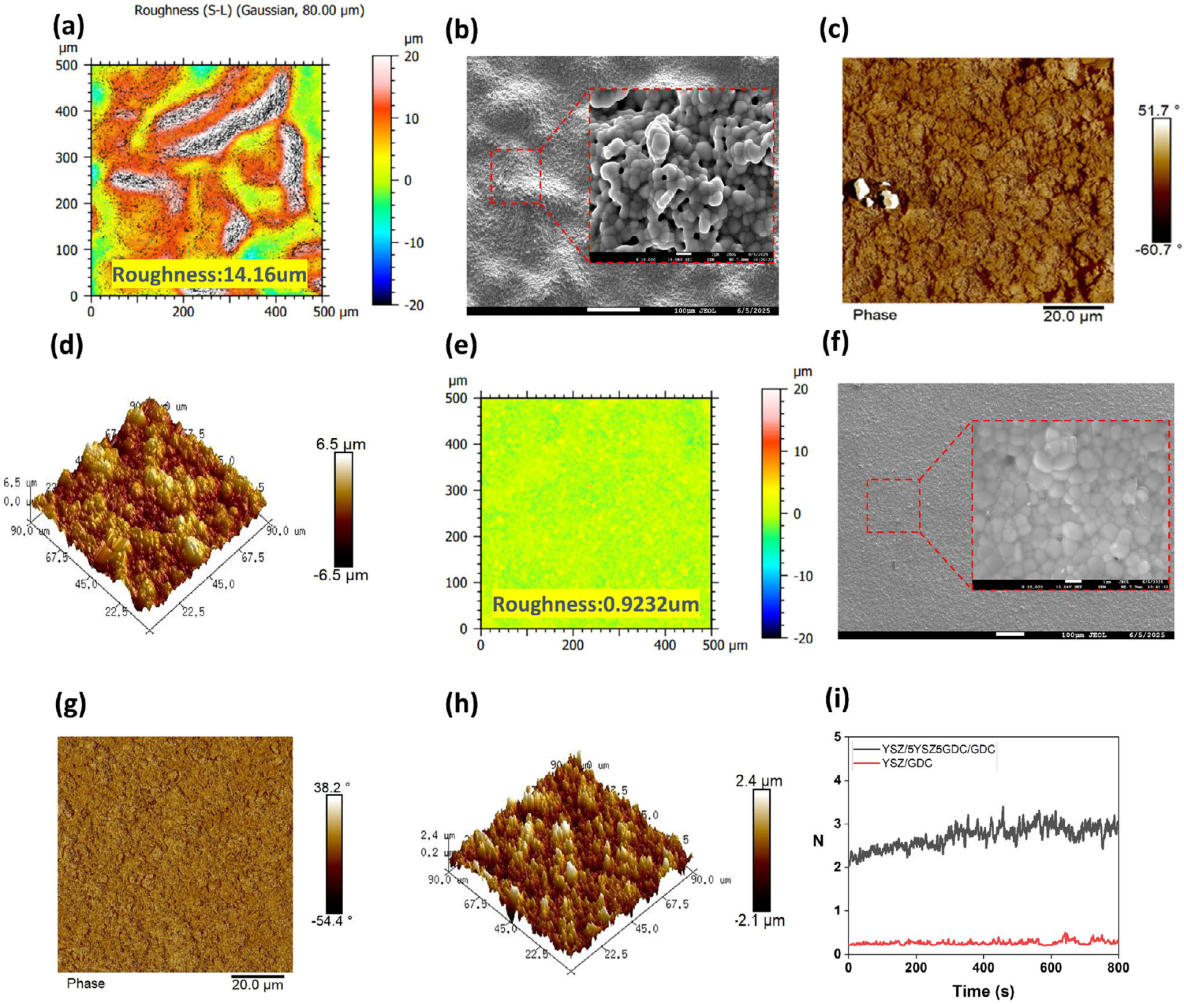
Surface analysis of GDC layer in YSZ/GDC electrolyte cells: a) confocal microscopy image, b) SEM image, c) AFM roughness image, and d) AFM height image. Surface analysis of GDC layer in YSZ/5YSZ5GDC/GDC electrolyte cells: e) confocal microscopy image, f) SEM image, g) AFM roughness image, and h) AFM height image. i) Delamination test of the electrolyte layer.

The influence of the composite electrolyte layer on adjacent electrolyte layers was investigated. In particular, the GDC electrolyte layer deposited on the outermost surface and directly interfacing with the oxygen electrode was analyzed. Surface imaging of the GDC layer was performed using confocal microscopy, SEM, and AFM. Figure S6 (Supporting Information) shows SEM images of the GDC electrolyte surface sintered at 900 °C, indicating similar morphologies and particle sizes regardless of the presence of an intermediate layer. However, after sintering at 1380 °C, at which the difference in thermal deformation disparity significantly affects material stability, the GDC surface in cells without the composite electrolyte exhibited instability and delamination. As confirmed by confocal microscopy, this resulted in a surface roughness of 15.86 µm over a 500 µm × 500 µm area. (**Figure** **4**a,b) AFM analysis revealed a large height variation (−6.5 to +6.5 µm), indicative of a highly heterogeneous surface composition or hardness distribution (Figure 4c,d). Conversely, the cells with the composite electrolyte layer demonstrated significantly reduced roughness (0.9232 µm) and small surface height variation (−2.1 to +2.4 µm), as shown in Figure 4e–h and Table S2 (Supporting Information), indicating a smoother GDC surface.

To quantitatively assess the interfacial bonding strength, a surface scratch test was conducted. Figure S7 (Supporting Information) illustrates the measurement process by which the force required to remove the electrolyte layer from the cell surface using a diamond blade was recorded. Experimental results showed that cells without a composite intermediate layer exhibited a weak adhesion strength of ≈0.25 N, whereas cells with the composite intermediate layer required ≈3 N of force to remove the electrolyte layer (Figure 4i). Based on the finding that the composite intermediate layer improves the interfacial bonding between YSZ and GDC, we carried out a detailed investigation. In particular, structural changes unique to the composite intermediate layer were systematically observed and analyzed.

### 3.3 Phase Formation and Oxygen Adsorption Enhancement in YSZ–GDC Composites

To closely examine the composite electrolyte structure, electrolyte‐type cells consisting solely of a composite electrolyte layer coated onto a YSZ substrate were fabricated. The crystalline structure of the composite intermediate layer was analyzed using X‐ray diffraction (XRD).

**Figure 5:**
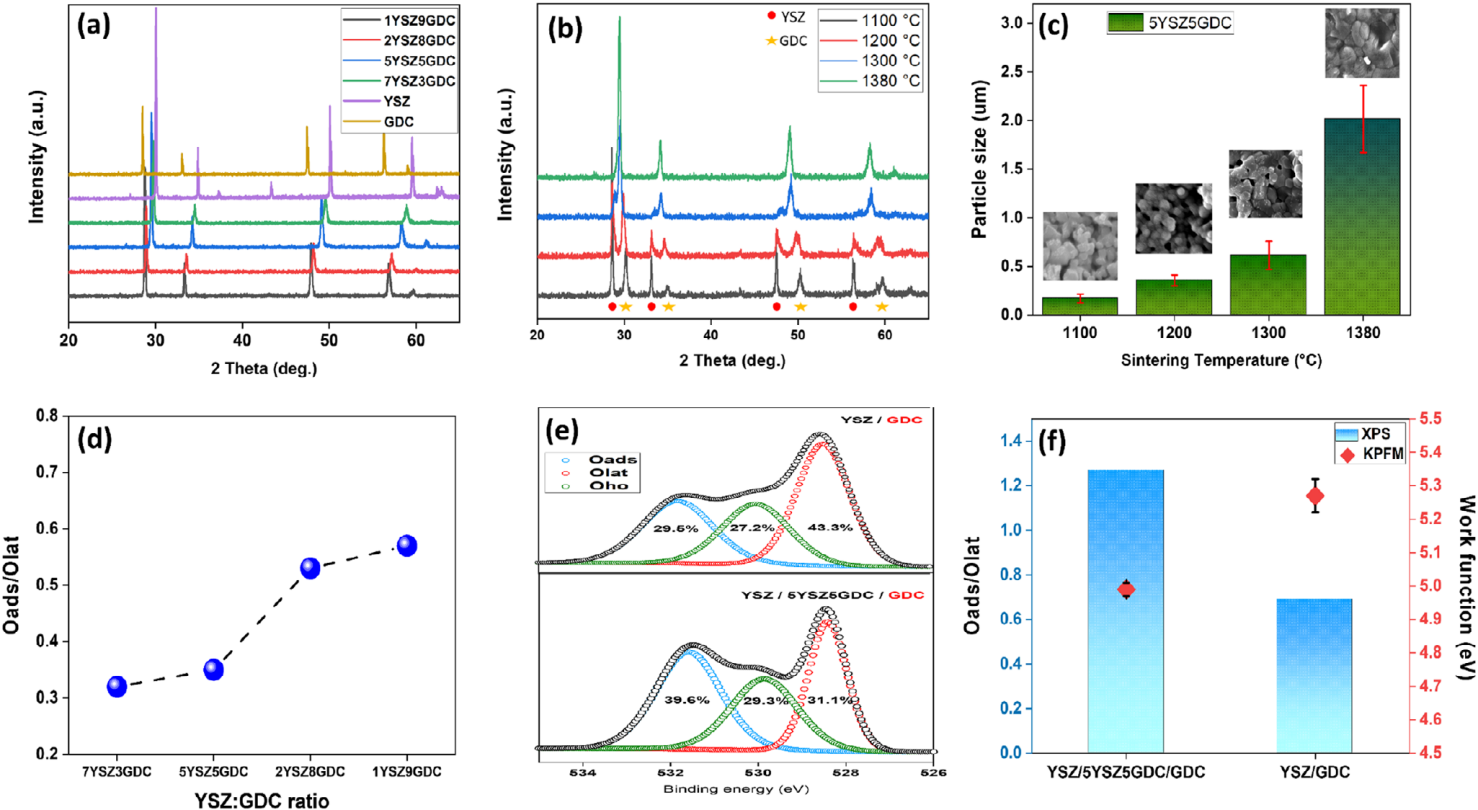
a) XRD patterns of composite electrolyte layers sintered at 1380 °C with varying YSZ:GDC ratios. b) XRD patterns of 5YSZ5GDC surface layer as a function of sintering temperature. c) Particle size of 5YSZ5GDC surface layer at different sintering temperatures. d) Oads/Olat ratios of composite electrolyte layer as a function of YSZ:GDC composition. e) XPS spectra of GDC surface for YSZ/GDC and YSZ/5YSZ5GDC/GDC electrolyte layers. f) Comparison of Oads/Olat ratio and work function values on GDC surface in YSZ/GDC and YSZ/5YSZ/GDC electrolyte structures.

The XRD patterns in **Figure** **5**a provide definitive evidence of single‐phase solid solution formation at 1380 °C. Quantitative phase analysis confirms that while pure YSZ maintains a cubic fluorite structure (Fm‐3m) with formula ZrO_2_._12_ (PDF 01‐081‐1550) and pure GDC exhibits CeO_2_ with the same structure (PDF 00‐034‐0394), the 2YSZ8GDC composite after 1380 °C sintering shows a single phase with formula (Zr_0_._2_Ce_0_._8_)O_2_ (PDF 01‐074‐8064), retaining the cubic fluorite structure.(Figure S8, Supporting Information) The Zr:Ce ratio of 0.2:0.8 precisely matches the initial 2:8 mixing ratio, demonstrating complete atomic‐level homogenization. The pattern at 1380 °C reveals a significant transformation: the distinct YSZ and GDC peaks merge into a single, unified set of peaks at intermediate 2θ values. Critically, the exact position of this merged peak systematically shifts with the YSZ:GDC ratio (Figure 5a), consistent with Vegard's Law.^[^ ^42^ ^]^ This compositional dependence is the classic signature of two materials mixing at the atomic level and definitively rules out the formation of an insulating secondary phase like pyrochlore‐structured Gd_2_Zr_2_O_7_, which would appear with space group Fd‐3m and at fixed 2θ positions regardless of the initial component ratio. Had such a phase appeared, characteristic superstructure peaks would be expected at 2θ values of ≈14°, 27°–28°, 37°, and 45°, none of which are present in our data.^[^ ^43^ ^]^ The resulting solid solution retains the highly desirable cubic fluorite structure of its parent materials, which is crucial for maintaining high oxygen ion conductivity.^[^ ^44^ ^]^ Additionally, we investigated changes in the crystalline structure of the composite intermediate layer at different sintering temperatures. Figure 5b demonstrates that as the sintering temperature increases, the intensity of peaks corresponding to the newly formed phases from the interaction between YSZ and GDC significantly increases. At the same time, the intensity of the individual YSZ and GDC peaks gradually diminishes. This phenomenon aligns with observations from the surface SEM images of the 5YSZ5GDC layer presented in Figure 5c and Figure S9 (Supporting Information). Particle aggregation and growth at elevated temperatures indicate the formation of a new phase arising from solid‐state bonding between YSZ and GDC. Although additional peaks emerged at 1300 °C, the persistence of distinct YSZ and GDC peaks suggests that a higher sintering temperature of 1380 °C is required to obtain a stable 5YSZ5GDC composite electrolyte layer. Figure 5d and Figure S10 (Supporting Information) illustrate that varying the YSZ:GDC ratio significantly influences the oxygen vacancy concentration on the composite electrolyte surface. The XPS O1s spectra typically exhibit three distinct peaks corresponding to lattice oxygen (Olat), high oxidation oxygen (Oho), and adsorbed oxygen (Oads).^[^ ^45^, ^46^, ^47^, ^48^ ^]^ The Oads species are particularly critical as they facilitate rapid electrochemical oxygen transport at the electrode. Consequently, a higher Oads ratio indicates easier oxygen adsorption on the electrode surface, thereby accelerating the reaction kinetics.^[^ ^49^ ^]^ A high Oads/Olat ratio particularly implies increased adsorption of oxygen ions at the electrode surface, subsequently enhancing oxygen ion transport into the electrolyte and improving ionic conductivity.^[^ ^50^ ^]^ Recent studies have also reported that the ratio between adsorbed oxygen and lattice oxygen can be used to effectively evaluate the catalytic activity of electrode materials.^[^ ^51^ ^]^ The Oads/Olat ratio was calculated using the integrated peak areas corresponding to adsorbed oxygen (Oads) and lattice oxygen (Olat) in the O1s XPS(Table S3, Supporting Information). The 1YSZ9GDC cell, containing the highest proportion of GDC, which exhibits superior oxygen ion adsorption capabilities compared to YSZ, showed the highest Oads/Olat value. Additionally, the single GDC electrolyte surface with 5YSZ5GDC interfacial layer exhibited an Oads/Olat ratio of 1.27. This value was significantly higher than the ratio of 0.67 observed for the GDC surface without an interfacial layer (Figure 5e; Table S4, Supporting Information).

To enhance the reliability of our XPS results and demonstrate that the observed chemical changes are rooted in fundamental structural differences rather than surface roughness artifacts, we introduced Raman spectroscopy (Figure S11, Supporting Information). This technique is ideal as it is highly sensitive to lattice defects, particularly to the concentration of oxygen vacancies (OVs) in ceria‐based materials. To ensure the reliability of the quantitative analysis, we adopted a normalization method by dividing the integrated area of the oxygen vacancy peak (A_OV, 565–590 cm^−1^) by that of the main F_2_g lattice vibration peak (A_F_2_g, 450–475 cm^−1^).^[^ ^52^ ^]^ This scientifically sound approach uses the F_2_g peak as an internal standard, which cancels out variations in signal intensity due to surface roughness and allows for a precise comparison of the intrinsic defect concentrations of the materials. The analysis revealed that the oxygen vacancy index (A_OV / A_F_2_g) for the GDC surface with the composite interlayer was 0.0142, significantly higher than the 0.0083 for the surface without it. This result provides direct and quantitative structural evidence that the composite interlayer induces a ≈71% higher concentration of surface oxygen vacancies.^[^ ^53^ ^]^ This increased defect concentration is made possible by the stable microstructure of the GDC layer formed on the composite interlayer (Figure 4f); the uniform grain size and high density of grain boundaries facilitate the formation of adsorbed oxygen species (Oads), which leads to the higher Oads/Olat ratio confirmed in our XPS analysis.^[^ ^54^, ^55^ ^]^ Furthermore, Kelvin Probe Force Microscopy (KPFM) was used to confirm the actual electrical consequences of these validated structural and chemical changes (Figure 5f; Figure S12, Supporting Information).^[^ ^56^, ^57^, ^58^ ^]^ The GDC surface, confirmed by Raman to exhibit stronger oxygen‐related features and by XPS to show a higher O_ads ratio, displayed a lower average work function (4.99 eV) compared to the reference surface (5.28 eV).^[^ ^57^ ^]^ This is in perfect agreement with the phenomenon where an increased concentration of oxygen vacancies and enhanced surface electron density raise the Fermi level, thereby reducing the work function.^[^ ^59^, ^60^, ^61^ ^]^ This fundamental enhancement of the surface properties is crucial in the actual operating environment of an SOEC. Given that the air electrode, lanthanum strontium manganite (LSM), has negligible oxygen ion conductivity, efficient oxygen ion oxidation and electron transfer at the well‐bonded GDC–LSM interface determine the cell's performance. By increasing the oxygen vacancy concentration and lowering the work function of the GDC surface through our proposed composite interlayer, we provide ideal conditions that promote the oxidation of oxygen ions (O^−2^) supplied from the electrolyte and facilitate O_2_ evolution. In conclusion, the experimental observation that the GDC coating surface is enhanced by the composite interlayer, characterized by a high Oads/Olat ratio and a low average work function, plays a decisive role in the interfacial behavior. These features significantly improve the interfacial bonding, oxygen ion conductivity, and electron emission, effectively reducing the interfacial resistance. Thus, the consistent results from three independent analytical techniques, Raman spectroscopy (structural defects), XPS (chemical state), and KPFM (electrical properties), robustly demonstrate that the composite interlayer is a key mechanism that expands the electrochemically active region and reduces polarization resistance, thereby enhancing both the performance and long‐term stability of the SOEC.

### 3.4 Electrochemical Performance and CO_2_ Electrolysis Stability of Composite Electrolyte Cells

**Figure 6:**
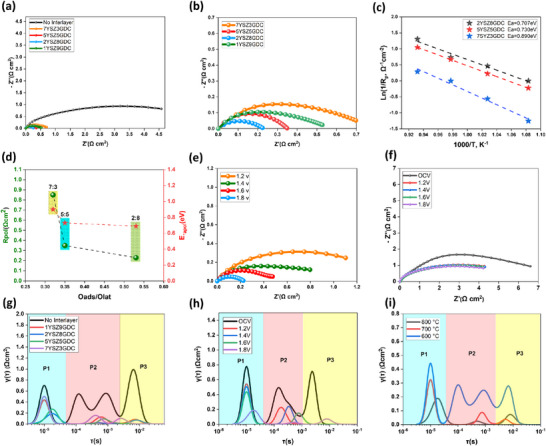
Electrochemical impedance spectroscopy analysis based on YSZ:GDC ratios of composite electrolyte layer with 50 sccm pure CO_2_ flow: a) Nyquist plots measured at 800 °C under 1.8 V. b) Detailed view of (a). c) Linear fit of Arrhenius plot for activation energy determination. d) Polarization resistance and activation energy as functions of Oads/Olat ratio of composite electrolyte layer. e) Voltage‐dependent Nyquist plots of cells with YSZ/2YSZ8GDC/GDC electrolyte layers. f) Voltage‐dependent Nyquist plots of cells with YSZ/GDC electrolyte layers. g) Distribution of relaxation time (DRT) analysis at 800 °C and 1.8 V according to varying composite electrolyte layer ratios. h) Voltage‐dependent DRT analysis for a cell with 2YSZ8GDC electrolyte layer at 800 °C. i) Temperature‐dependent DRT analysis for a cell with 2YSZ8GDC electrolyte layer at 1.8 V.

Electrochemical impedance spectroscopy (EIS) was conducted to evaluate the influence of structural and chemical properties of cells containing composite electrolyte layers on electrochemical reactions. All samples were systematically prepared as single cells, enabling a comprehensive performance comparison compatible with fuel electrode‐supported single‐cell

configurations. Nyquist plots (**Figure** **6**a,b) measured at 800 °C and 1.8 V demonstrate that cells incorporating composite electrolyte layers exhibited lower polarization resistance (Rp) than did those without composite layers. Notably, the cell incorporating a 2YSZ8GDC intermediate layer exhibited the lowest polarization resistance (Rp) of 0.22 Ω·cm^2^ at 800 °C, representing a substantial reduction compared to the reference cell. Interestingly, although the 1YSZ9GDC cell exhibited the highest Oads/Olat ratio, it showed higher Rp values than both 2YSZ8GDC and 5YSZ5GDC. This was attributed to the insufficient YSZ content, which led to weaker interfacial bonding and structural defects that significantly increased resistance. The notable reduction in Rp values observed with composite intermediate layers suggests that additional factors beyond increased interfacial area contribute to resistance reduction. As shown in Figure 6c and Figure S13 (Supporting Information), variations in composite intermediate layer composition altered activation energies, indicating that electrochemical reactivity is sensitive to the chemical composition of the intermediate layer. The extended interfacial structure formed by electrolyte components within the composite intermediate layer increases the reactive surface area. In addition, it facilitates the oxidation reaction at the LSM–GDC interface, thereby enhancing the overall electrochemical activity. In particular, the composite electrolyte layer improves the interfacial properties of GDC, simultaneously strengthening interfacial adhesion with LSM and increasing electrochemical reactivity. This structure enhances oxygen‐ion conductivity and promotes the formation of adsorbed Oads, thus enabling more efficient reactions of oxygen ions (O^−2^) and electrons (e^−^) at the LSM–GDC interface. Specifically, as the GDC content increases, Oads formation and oxygen‐ion mobility are significantly enhanced, more strongly activating oxidation reactions at the LSM–GDC interface compared to the case of pure YSZ interfaces, and substantially improving the overall electrochemical performance. A comparison of ratios of 7:3, 5:5, and 2:8, which were structurally intact, showed clear trends in which higher Oads/Olat ratios corresponded to lower activation energies and Rp values (Figure 6d). These findings suggest that increasing GDC content critically enhances Oads, driving oxygen ion transport reactions at the electrode surface and confirming the Oads/Olat ratio as a crucial indicator of electrochemical reactivity. Therefore, the remarkable reduction in polarization resistance (Rp) can be attributed to the synergistic effects of three key factors: 1) the higher intrinsic ionic conductivity of GDC, which enhances oxygen‐ion transport within the composite interlayer; 2) the formation of a (Zr,Ce)O_2_ solid solution at high temperature, providing a coherent lattice bridge that lowers interfacial energy barriers; and 3) the 3D interlocking microstructure, which extends the effective YSZ–GDC interfacial area from a 2D plane to a 3D active volume, thereby accelerating ionic transfer and interfacial reactions (Rp ∝ 1 / (Active Reaction Area).^[^ ^42^, ^62^, ^63^ ^]^ These combined factors offer a clear and quantitative explanation for the substantial Rp reduction observed in the composite electrolyte cells. Furthermore, to investigate the dynamic behavior and electrochemical responses influenced by composite intermediate layers, distribution of relaxation time (DRT) analysis was performed. The DRT analysis distinguished overlapping electrode processes and identified reaction contributions in EIS data. In this study, a Python‐based DRT program developed by the MIT Ciuccislab group was employed. Analyzing the results of the Distribution of Relaxation Time (DRT), three distinct regions (P1–P3) were observed, starting from the lowest relaxation time. Each region corresponded to different electrochemical processes related to electrode reactions, with the integral area representing the associated resistance(Table S5a, Supporting Information).^[^ ^64^, ^65^ ^]^ The processes contributing to region P1 were related to oxygen ion transport across the electrode/electrolyte interface and subsequent incorporation into the electrolyte.^[^ ^66^ ^]^ Notably, the 2YSZ8GDC composition showed the lowest resistance in region P1 (Figure 6g; Table S5b, Supporting Information), attributed to its highest proportion of GDC, which exhibits superior oxygen ion conductivity and maintains structural integrity without cracks compared to other ratios. Region P2 corresponded to the process of oxygen ion transport within the electrode. Introducing the composite intermediate layer notably promoted oxygen ion diffusion and integration at the electrode‐electrolyte interface, significantly reducing the corresponding intermediate peak.^[^ ^67^ ^]^ Region P3 was closely associated with CO_2_ adsorption and electrochemical surface reactions (fuel gas conversion). Although no clear trend was observed regarding different composite intermediate layer ratios, all composite‐containing cells exhibited substantially reduced resistance compared to the reference cell. This reduction in resistance arose because the electrolyte layers without composite layers (YSZ and GDC) exhibited structural instability and cracking, impairing oxygen ion transfer to the air electrode and hindering electron supply for the CO_2_ reduction reaction (CO_2_RR). Consequently, unreacted CO_2_ accumulated within the electrode, reducing the gas concentration gradient and slowing gas flow, manifesting as increased resistance peaks in region P3 of the DRT.^[^ ^68^, ^69^ ^]^

**Figure 7:**
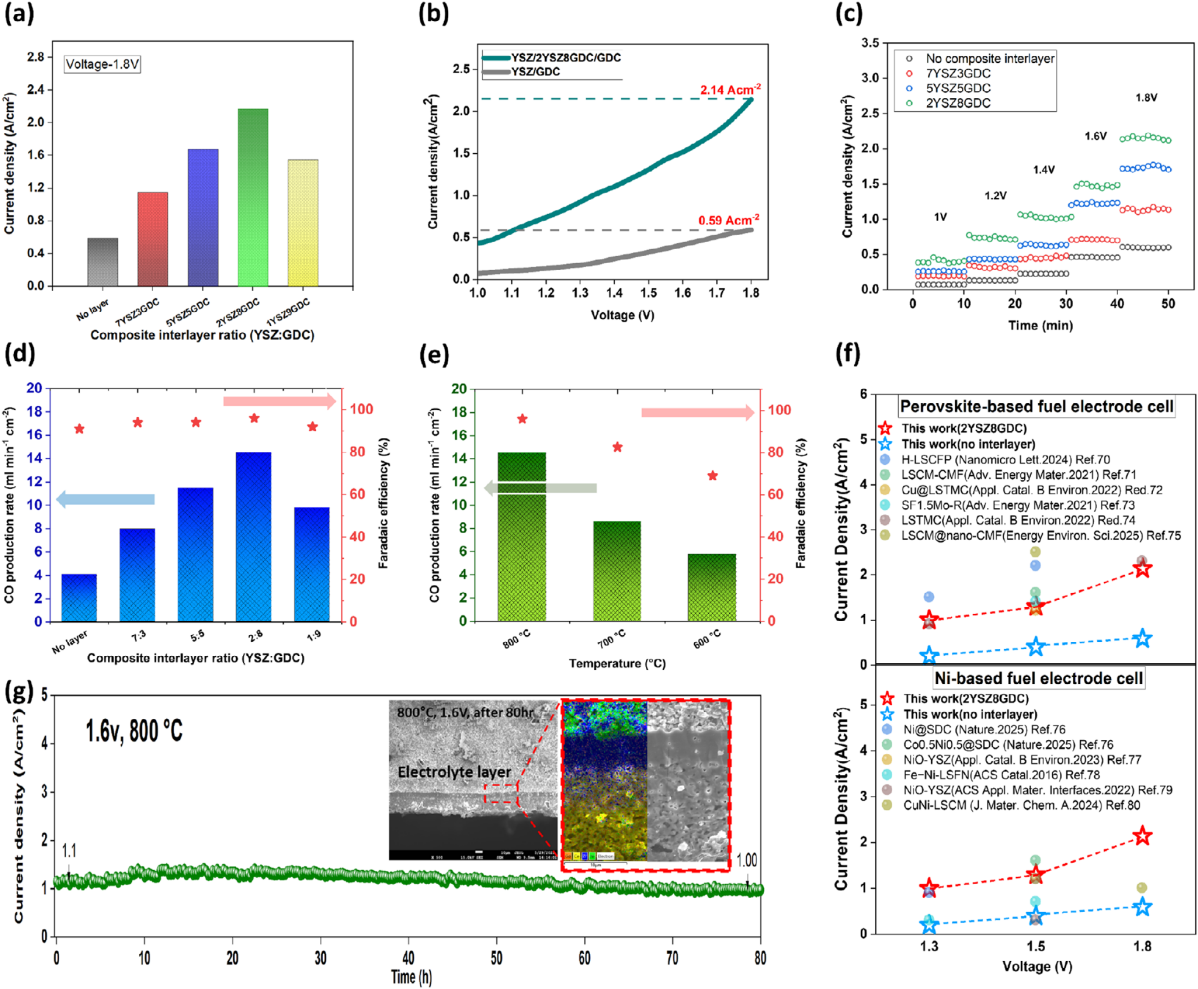
Evaluation of electrochemical performance and stability of cells with electrolyte composite interlayers: a) Current density at 1.8 V as a function of composite electrolyte layer ratio. b) I‐V curves comparing cells with and without composite electrolyte layers. c) Potentiostatic tests of corresponding cells. d) Faradaic efficiency and CO production rate during CO_2_ electrochemical reduction reaction (CO_2_RR) in SOEC cells with composite electrolyte interlayers at an applied potential of 1.8 V in 100% CO_2_ gas flowing at 50 sccm. e) Temperature‐dependent CO production rate and Faradaic efficiency. f) CO_2_ electrochemical reduction performance comparison with Ni‐ and perovskite‐based fuel electrodes at various voltages. g) Stability test of 5YSZ5GDC cell at 800 °C and 1.6 V with SEM images after long‐term operation.

Moreover, to explore the effects of voltage on CO_2_RR, impedance spectra under various voltages at 800 °C were investigated using cells without composite layers and cells with the 2YSZ8GDC electrolyte layer, as depicted in Figure 6e–h. Particularly, Figure 6e,h, and Table S5c (Supporting Information) demonstrate a substantial decrease in polarization resistance across the entire frequency range with increasing applied voltage for the cell containing the 2YSZ8GDC electrolyte layer. The significant reduction in polarization resistance with increasing polarization voltage aligns well with previous studies indicating that applied voltage effectively activates the electrode.^[^ ^68^ ^]^ Furthermore, Figure 6i and Table S5d (Supporting Information) indicate that decreasing temperature reduces oxygen ion mobility and increases internal electrolyte resistance, thereby elevating polarization resistance throughout the measured frequency range. CO_2_ electrolysis was performed using a single cell consisting of a NiO+YSZ cathode and a YSZ–GDC composite electrolyte, with pure CO_2_ supplied to the cathode and the anode exposed to ambient air. **Figure** **7**a presents the maximum current density of the NiO+YSZ‐based single cells with varying ratios of the composite electrolyte layer, which exhibited a trend similar to that observed in the interfacial polarization resistance (Rp). At 800 °C, the maximum current density for the 2YSZ8GDC cell was measured at 2.14 A cm^−2^ at 1.8 V, whereas the cell without an interfacial layer reached a value of only 0.59 A cm^−2^. These results clearly demonstrate that the composite electrolyte layer significantly enhances the electrolysis performance of NiO+YSZ‐based cathodes in SOEC applications. Figure 7b shows *I–V* curves for direct CO_2_ electrolysis of the highest‐performing 2YSZ8GDC cell at 800 °C. Additionally, operational stability for CO_2_ electrolysis at different composite electrolyte layer ratios was evaluated at 800 °C by measuring current density over time under various voltage conditions (Figure 7c). Apart from stable operation, the current density increased with rising applied voltage. During electrochemical stability assessment, exhaust gases were collected, and the CO proportion was analyzed using gas chromatography. Figure 7d illustrates the CO production rates and corresponding Faradaic efficiencies at different YSZ:GDC electrolyte ratios. Notably, the CO production rate increased up to the 2:8 (YSZ:GDC) ratio but decreased at the 1:9 ratio. This decline at the 1:9 ratio aligns with previous results indicating electrolyte cracking, increased resistance, and reduced current density, consequently lowering CO production rates. Furthermore, temperature‐dependent measurements of CO production rates and Faradaic efficiencies revealed decreases at lower temperatures (Figure 7e). The electrolyte's ionic conductivity is temperature‐sensitive; as shown by the DRT analysis in Figure 6i, reduced temperatures slowed O^−2^ ion transport and increased internal electrolyte resistance, leading to a decline in CO production rates. Additionally, it was observed that the Faradaic efficiency decreased at lower temperatures due to increased carbon deposition through the Boudouard reaction (CO + CO → CO_2_ + C) occurring in electrode environments containing CO. The maximum current density achieved during the test was compared with the highest values reported in the literature (Figure 7f). The cell with the composite electrolyte layer showed performance comparable to that of perovskite‐based systems and the highest output among Ni‐based electrodes.^[^ ^70^, ^71^, ^72^, ^73^, ^74^, ^75^, ^76^, ^77^, ^78^, ^79^, ^80^ ^]^ Building on the widely adopted NiO–YSZ structure, further optimization of the Ni‐based electrode with the composite layer is expected to enable high electrochemical performance and scalable large‐area cell development. To further evaluate the long‐term stability and electrolyte interfacial adhesion, experiments were conducted under a high‐voltage condition of 1.6 V; the results are presented in Figure 7g. The single cell with the composite electrolyte layer retained ≈91% of its initial performance after 80 h of continuous operation. Notably, it exhibited excellent stability even under a high applied voltage of 1.6 V, which, to allow a rigorous evaluation of the delamination resistance of the electrolyte layer, is intentionally higher than those typically used in long‐term tests reported in previous studies.^[^ ^68^, ^69^, ^81^, ^82^ ^]^ Moreover, the SEM images in Figure 7g confirm that the electrolyte layer remained firmly adhered without any delamination, demonstrating robust interfacial stability even after 80 h of high‐voltage operation.

## 4 Conclusion

In this study, a YSZ‐GDC composite intermediate electrolyte layer was designed and systematically evaluated for CO_2_ electrolysis to address the issues of thermal mismatch and weakened interfacial adhesion between YSZ and GDC in single‐cell SOEC configurations. Composite layers with various compositions (YSZ:GDC ratios of 7:3, 5:5, 2:8, and 1:9) were successfully implemented, showing excellent interfacial adhesion without delamination even after sintering at 1380 °C. This outcome was attributed to the formation of a stable (Zr,Ce)O_2_ solid solution, which mitigated thermal deformation disparity. Furthermore, the composite interlayer enhanced the GDC surface by increasing surface oxygen vacancies. This synergistically improved oxygen adsorption (Oads/Olat ratio) and lowered the surface work function, thereby boosting interfacial reactivity and stability. Electrochemical characterization demonstrated that cells incorporating the composite intermediate layer exhibited significantly reduced polarization resistance (Rp) compared to conventional cells. The SOEC cell employing the engineered composite interlayer achieved a maximum current density of 2.14 A cm^−2^ and a minimum polarization resistance (Rp) of 0.22 Ω cm^2^ at 800 °C, surpassing the performance of previously reported Ni‐based fuel electrode cells under comparable conditions. These results clearly demonstrate the effectiveness of interface engineering in significantly enhancing electrochemical performance. Furthermore, the performance degradation was limited to within 9% after 80 h of high‐voltage operation (1.6 V), with sustained interfacial adhesion and superior long‐term stability. These findings demonstrate that the composite electrolyte layer serves as a robust and scalable platform technology for the commercialization of mechanically stable, large‐area SOECs. By effectively addressing the critical challenge of interfacial delamination during high‐temperature co‐sintering, this approach ensures superior structural integrity and reliability. Building on the exceptional interfacial stability achieved in this study, future work will focus on optimizing the electrolyte thickness to further reduce ohmic resistance through advanced thin‐film fabrication techniques such as ultrasonic spray coating, thereby paving the way for high‐performance, large‐area Ni‐based solid oxide electrolysis cells.

## Conflict of Interest

The authors declare no conflict of interest.

## Supporting information



Supporting Information

## Data Availability

The data that support the findings of this study, including raw electrochemical measurements (EIS, *I–V* curves, and DRT analyses), microstructural characterization data (SEM, XRD, AFM, Raman, Dilatometry, and XPS), and long‐term stability test results, are available from the corresponding author upon reasonable request. Additional processed data presented in the figures and tables of this article are included in the published manuscript and Supplementary Information.
