# Supplementary material for: High‐Efficiency CO2 Electrolysis Enabled by Interface‐Engineered Composite Electrolytes in Ni‐Based SOEC
Source: Adv Sci (Weinh). 2025 Dec 8;13(14):e18091. doi: 10.1002/advs.202518091 (PMC12970289; doi:10.1002/advs.202518091)
Supplement: Supplementary file 1 — Supporting Information [file ADVS-13-e18091-s001.docx]

Supporting Information

**High-Efficiency CO₂ Electrolysis Enabled by Interface-Engineered Composite Electrolytes in Ni-Based SOEC**

*Rustam Yuldashev, ^1,2^* *Hyunchul Jung, ^1^* *Ji Hoon Park,^1,2,^* Jin Hee Lee,^1,2,^* and*

Min-Chul Kim, ^1,^*

**Figure S1.**

a) Photographs of dip-coating solutions for YSZ, 5YSZ5GDC, and GDC.

**
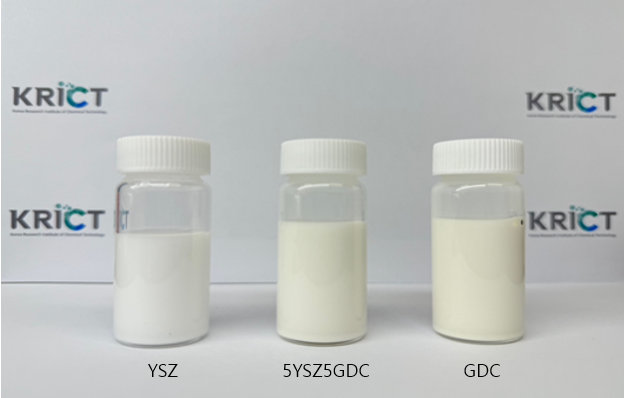
**

b) Images of cells at various fabrication stages.


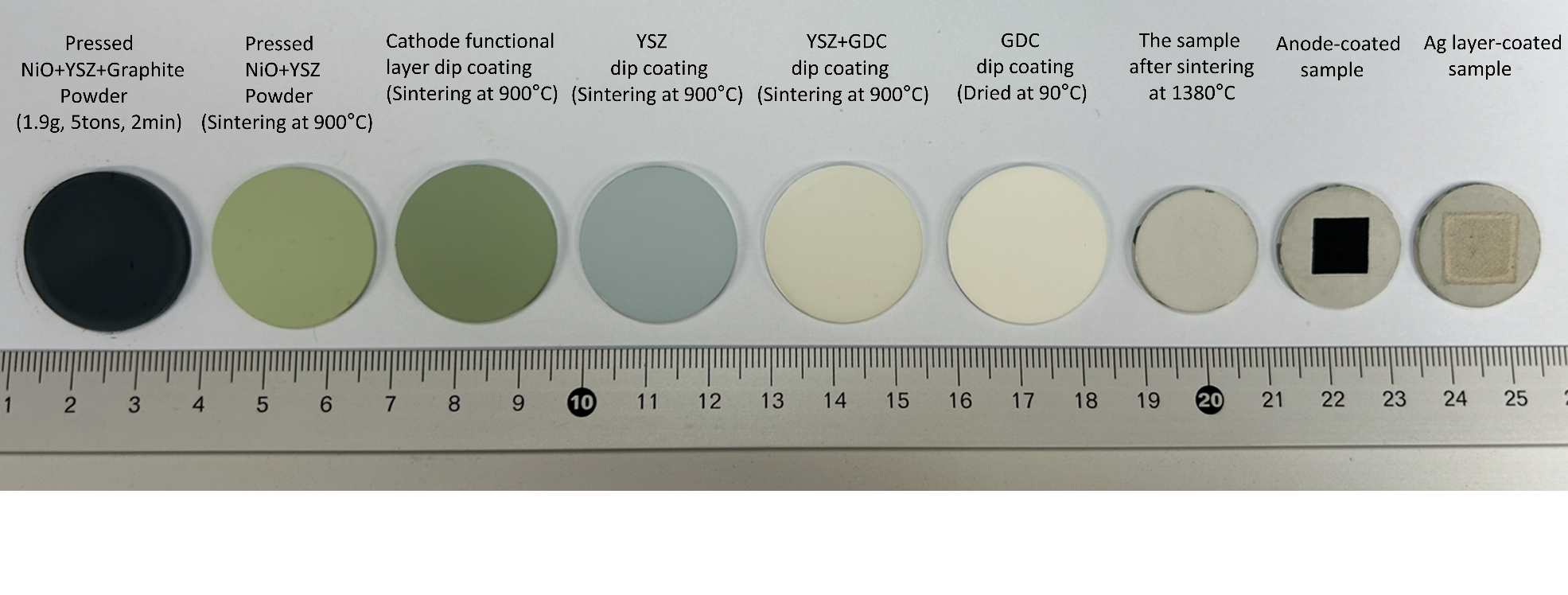


**Figure S2.** CO calibration fitting curve for quantification of produced gases using gas chromatography (GC).

1. CO 0ml/min: CO_2_ 50ml/min
2. CO 6.25ml/min: CO_2_ 43.75ml/min
3. CO 12.5ml/min: CO_2_ 37.5ml/min
4. CO 25ml/min: CO_2_ 25ml/min
5. CO 50ml/min: CO_2_ 0ml/min

**Figure S3.** a) SEM image and b) elemental mapping analysis of YSZ/GDC electrolyte layer after sintering at 900 °C.


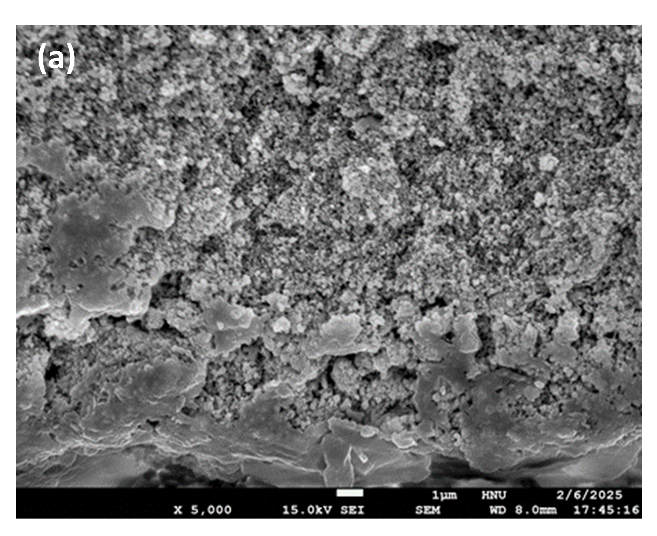

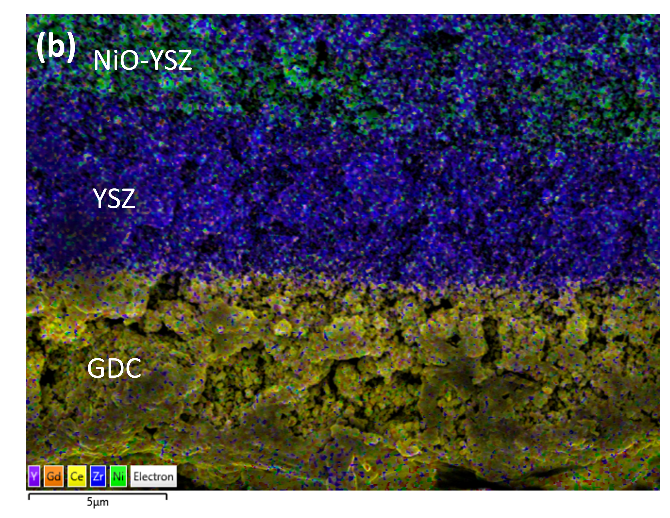


**Figure S4.** SEM and elemental mapping analyses of electrolyte layers after sintering at 1380 °C: a) YSZ/GDC and b) YSZ/5YSZ-5GDC/GDC structures.


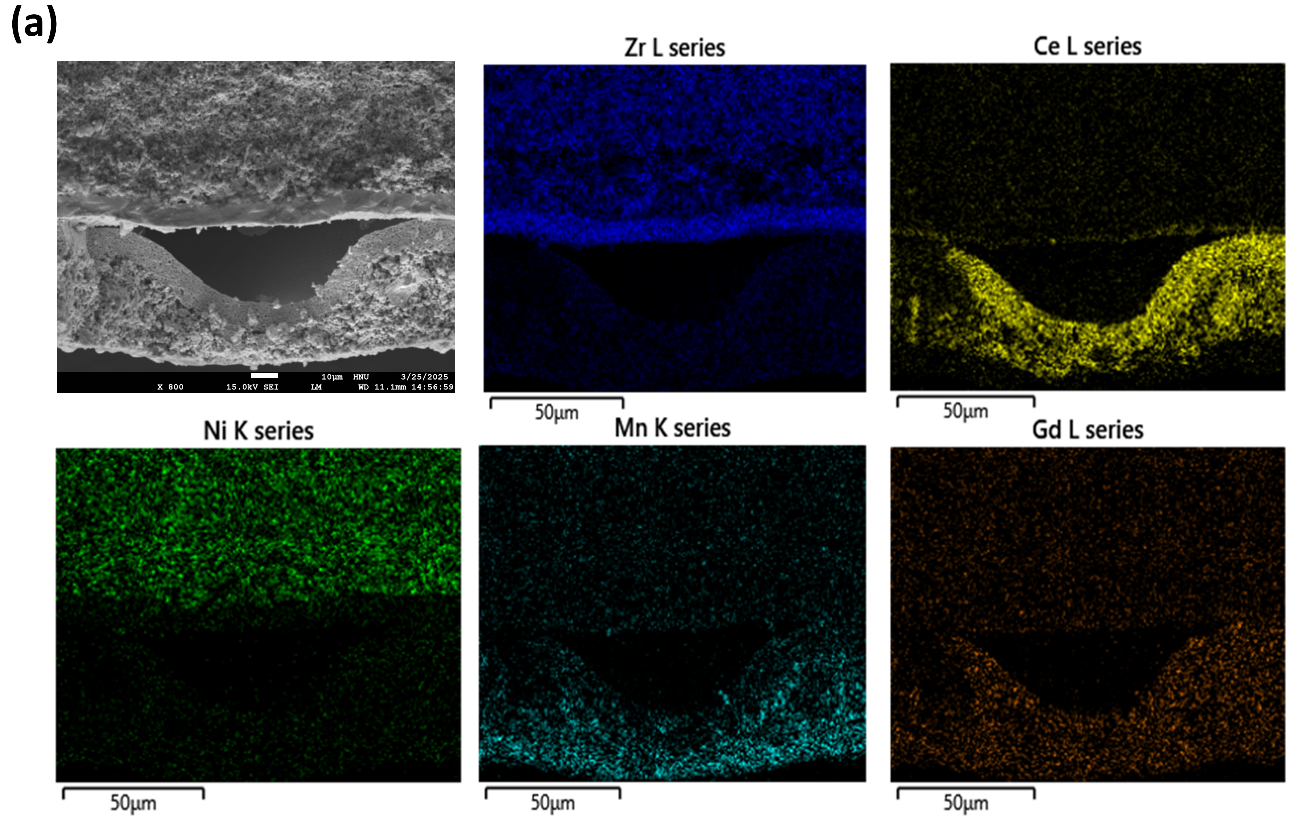


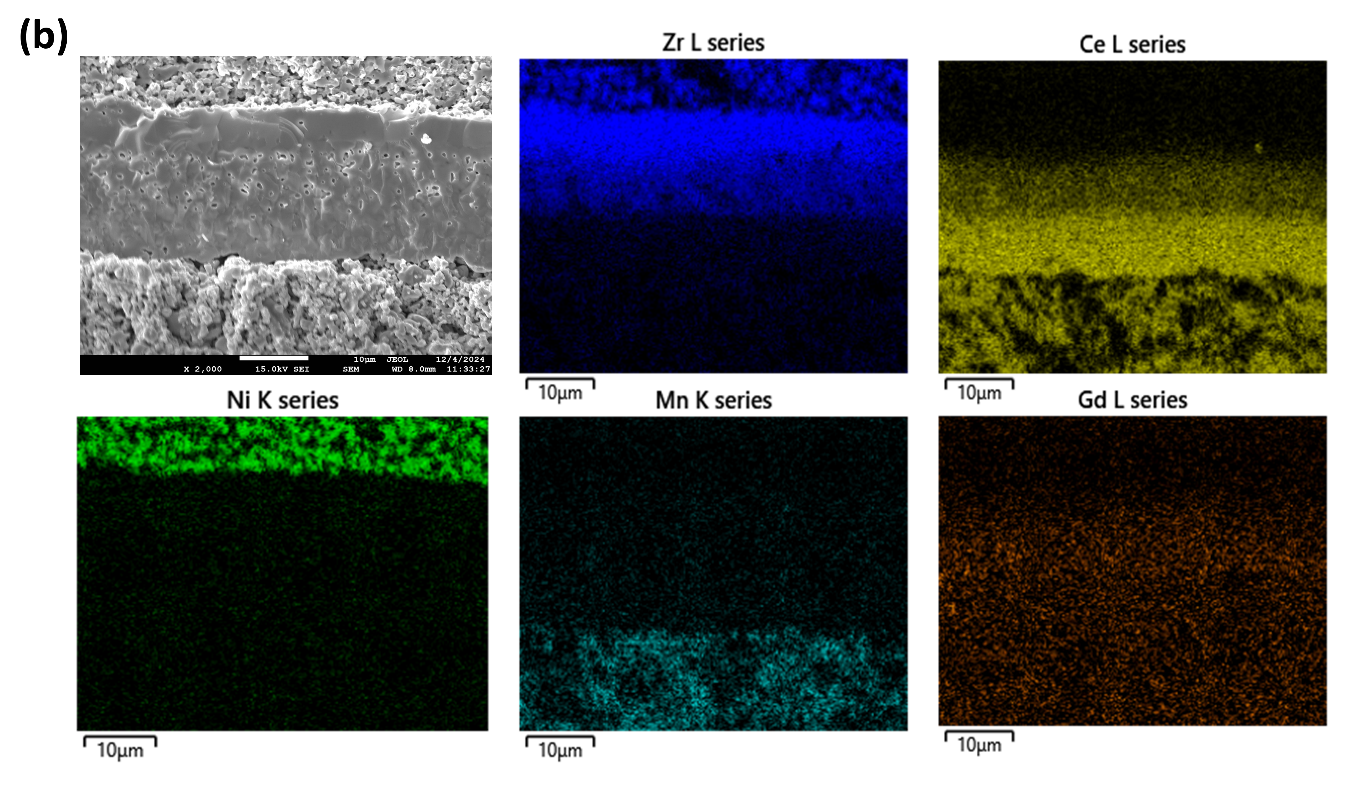


**Figure S5.** Thermal deformation behavior of GDC, YSZ, and 5YSZ5GDC composite.


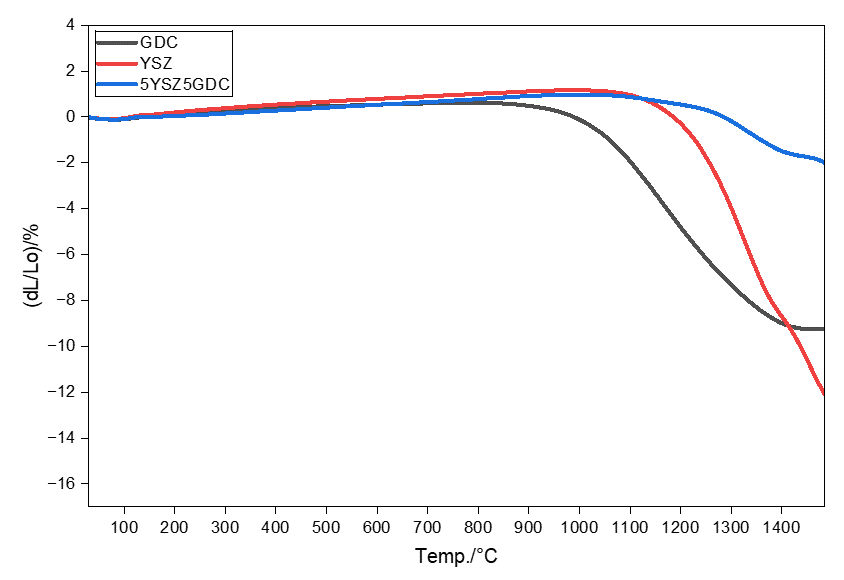


**Table S1.** Weight-to-volume conversion and percolation analysis of YSZ-GDC composite intermediate layers. Volume fractions were calculated based on the densities of YSZ (6.05 g/cm³) and GDC (7.22 g/cm³).

| Composition | Weight Ratio [YSZ:GDC] | YSZ Volume Fraction [vol%] | GDC Volume Fraction [vol%] | Percolation Status |
| --- | --- | --- | --- | --- |
| 7YSZ3GDC | 7:3 | 73.6 | 26.4 | Above threshold (continuous network) |
| 5YSZ5GDC | 5:5 | 54.4 | 45.6 | Above threshold (continuous network) |
| 2YSZ8GDC | 2:8 | 23 | 77 | Near threshold (semi-continuous) |
| 1YSZ9GDC | 1:9 | 11.7 | 88.3 | Below threshold (isolated domains) |

**Figure S6.** SEM images of GDC electrolyte layer after calcination at 900 °C: (a) Surface of GDC (YSZ/GDC) electrolyte layer, and (b) surface of GDC (YSZ/5YSZ5GDC/GDC) electrolyte layer.

(a)


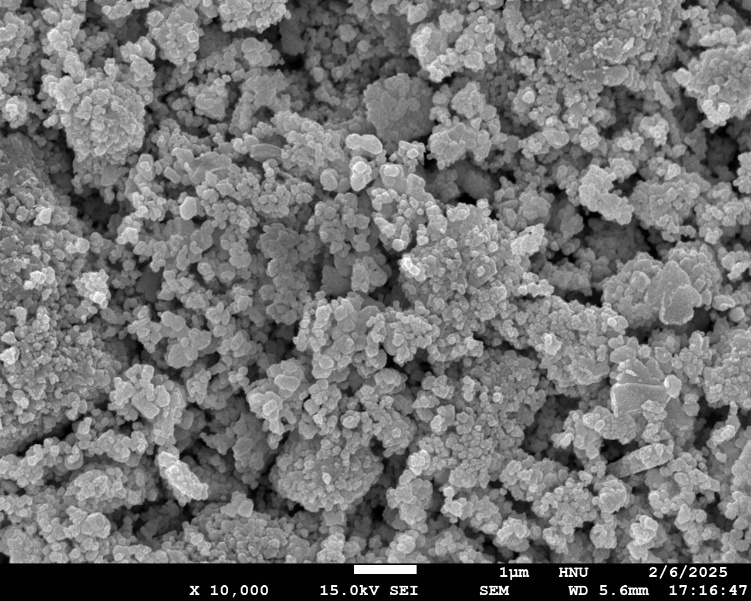


(b)


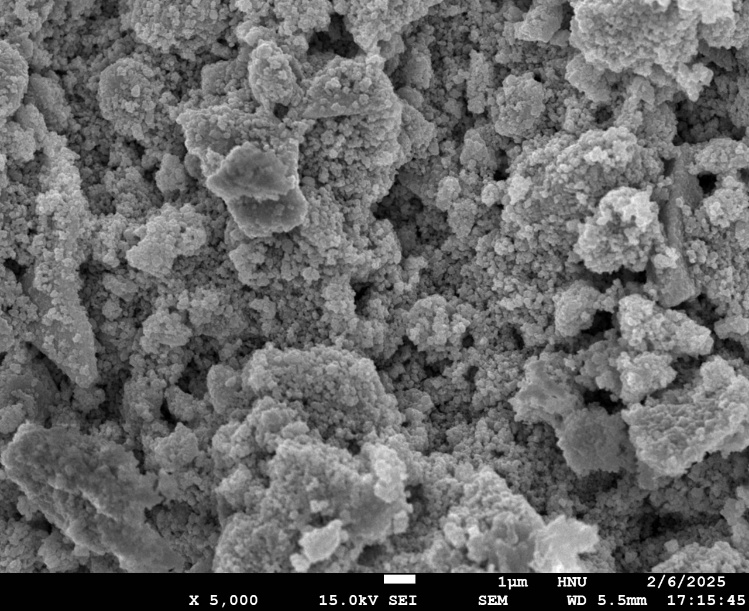


**Table S2.** Surface roughness parameters of two samples obtained via 3D optical profilometry based on ISO 25178 standard. Surface A exhibits moderate roughness and a relatively symmetric height distribution, while Surface B shows significantly higher roughness with pronounced valleys (negative skewness) and higher peak density. These characteristics may affect surface coating or printing uniformity.

| Parameter | Unit | Surface A  [GDC layer in  YSZ/5YSZ5GDC/GDC electrolyte cells] | Surface B  [GDC layer in YSZ/GDC electrolyte cells] | Description |
| --- | --- | --- | --- | --- |
| **Sa** | μm | 0.9232 | 15.86 | Arithmetic mean  height |
| **Sq** | μm | 1.219 | 24.05 | Root mean square height |
| **Sp** | μm | 88.71 | 151.8 | Maximum peak  height |
| **Sv** | μm | 51.18 | 129.7 | Maximum valley  depth |
| **Sz** | μm | 139.9 | 281.5 | Maximum height  (Sp + Sv) |
| **Ssk** | – | 0.1980 | -2.394 | Skewness |
| **Sku** | – | 17.99 | 9.152 | Kurtosis |
| **Spd** | 1/μm² | 0.000764 | 0.0224 | Peak density |
| **Sk** | μm | 2.806 | 17.71 | Core roughness  depth |

**Figure S7.** a) Photograph of SAICAS-EX system used to evaluate interfacial adhesion strength.
b) 1 mm-wide diamond blade (Dia blade) employed for surface scratching of thin film.
c) Observed delamination at interface of cell with YSZ/GDC electrolyte layer.
d) Observed delamination at interface of cell with YSZ/5YSZ5GDC/GDC electrolyte layer.

1.
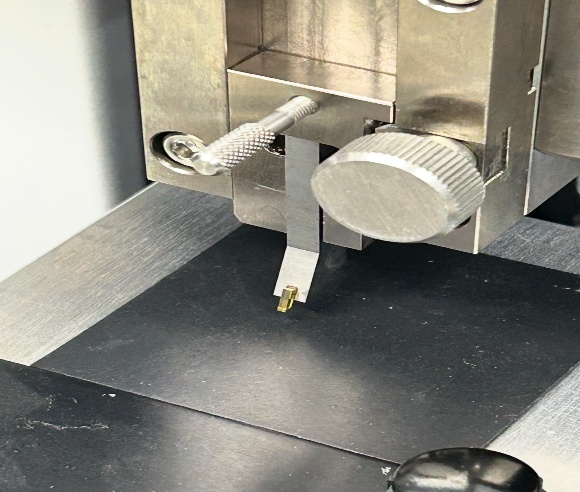

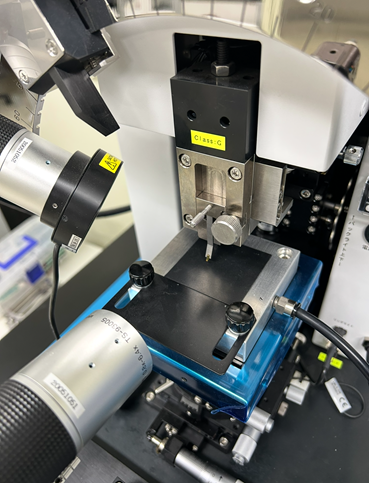
 b)


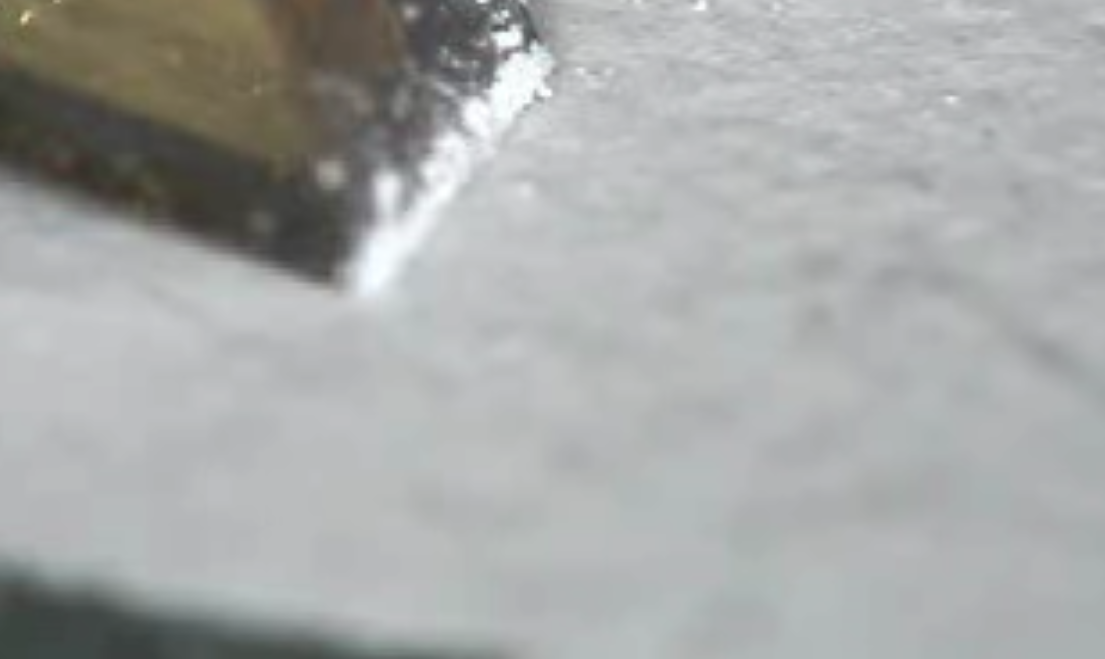

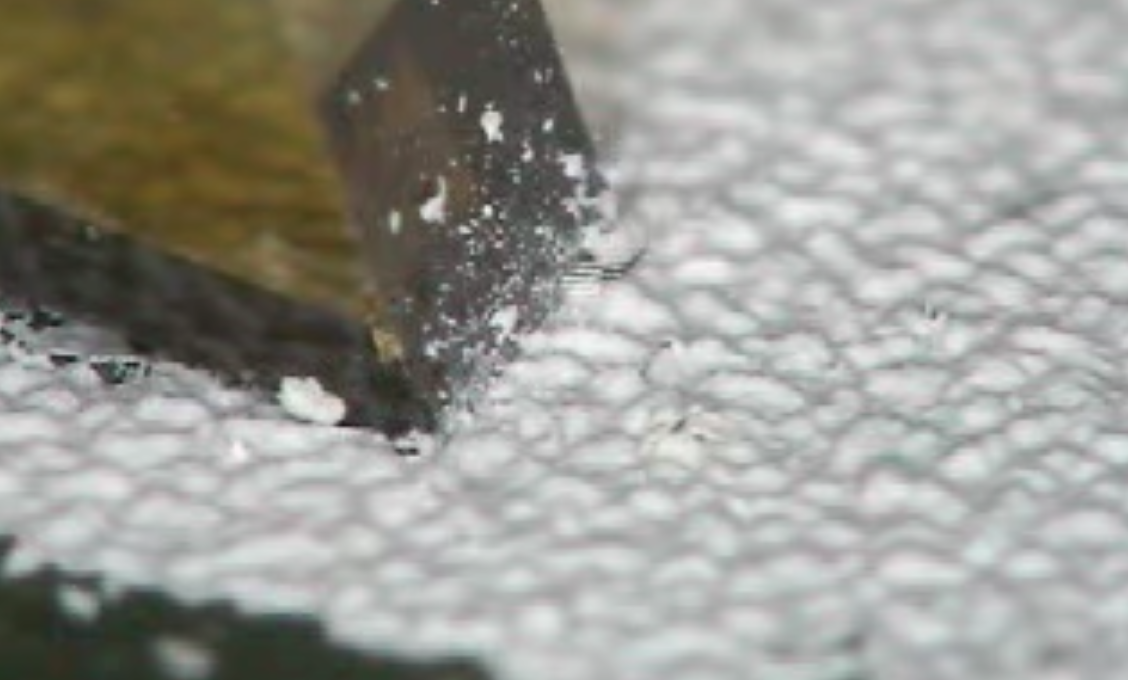


c) d)

**Figure S8. XRD Phase Analysis of Dip-Coated YSZ, GDC, and 2YSZ8GDC Composite Electrolyte Layers Sintered at 1380°C**

**
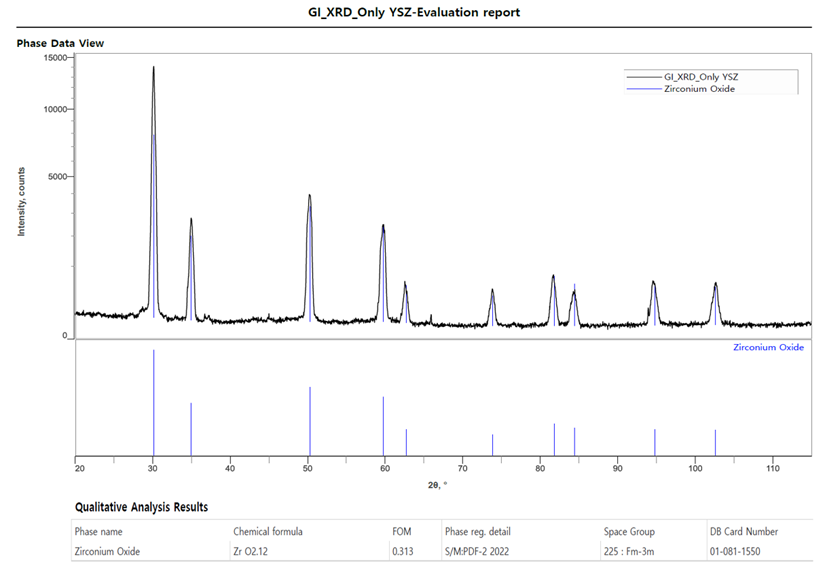
**

Figure S8a XRD Phase Analysis of Dip-Coated Pure YSZ Layer Sintered at 1380°


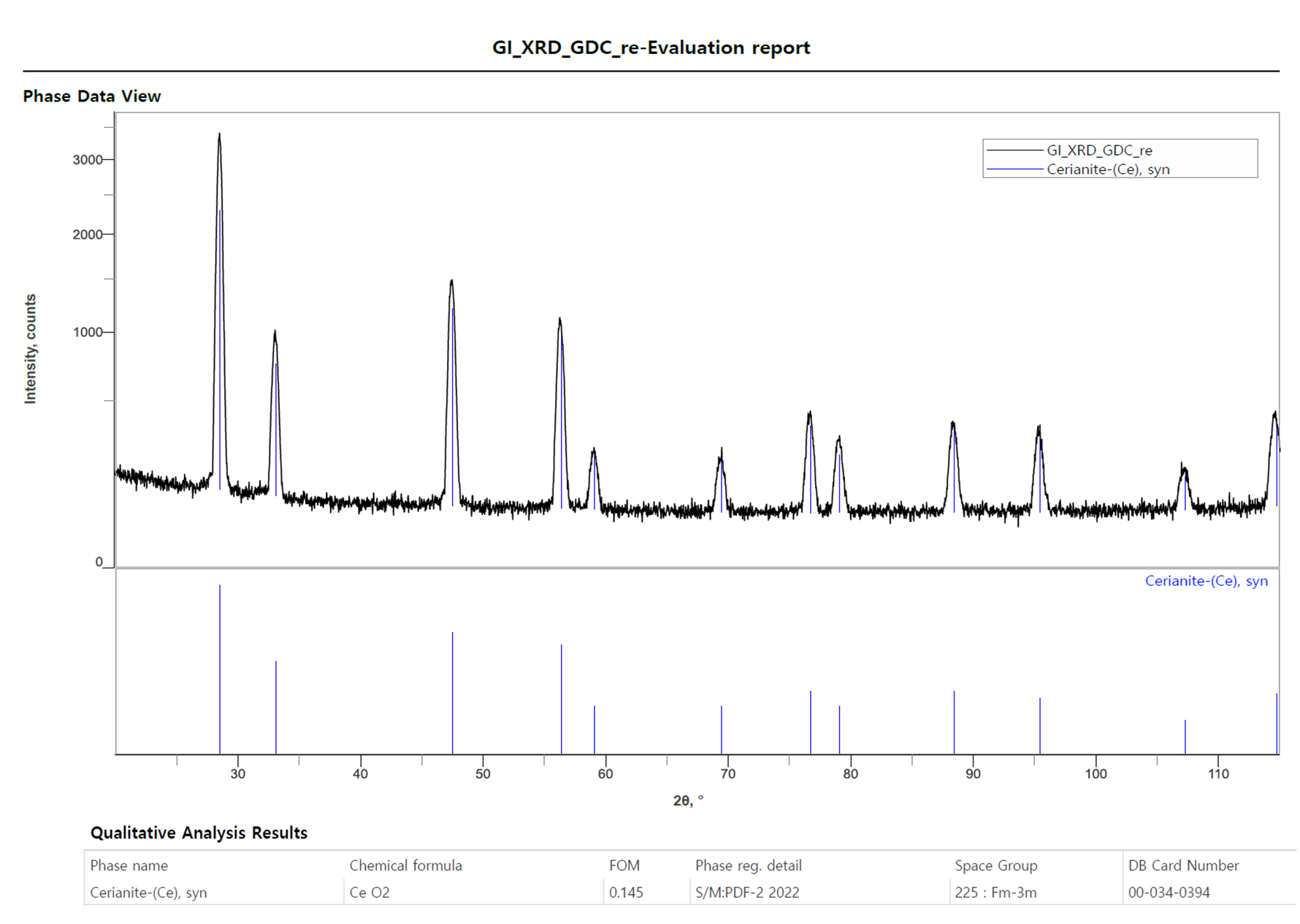


Figure S8b XRD Phase Analysis of Dip-Coated Pure GDC Layer Sintered at 1380°


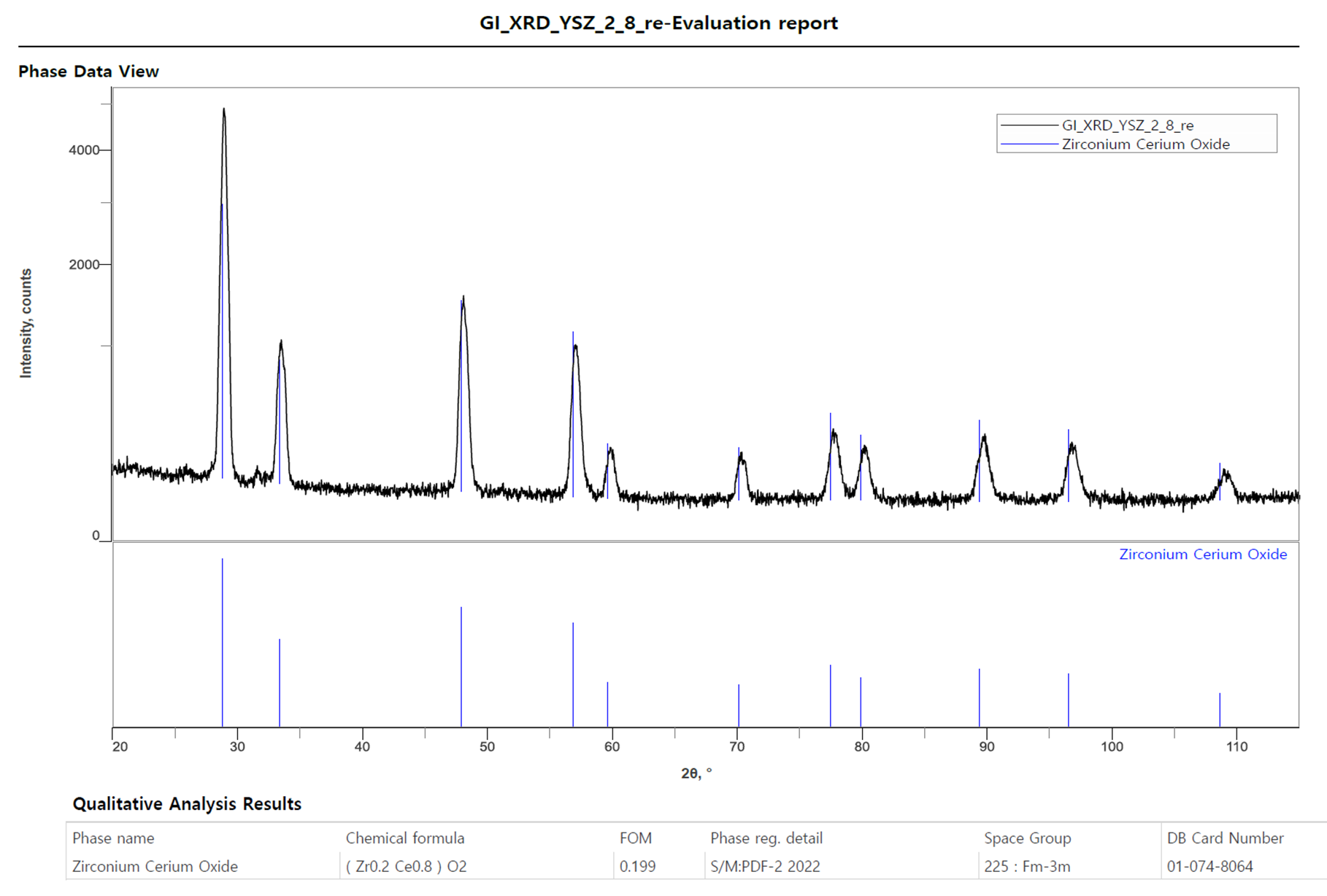


Figure S8c XRD Phase Analysis of Dip-Coated 2YSZ8GDC Composite Electrolytes After 1380°C Sintering

**Figure S9.** SEM images and elemental mapping of 5YSZ5GDC electrolyte surface measured at different sintering temperatures.


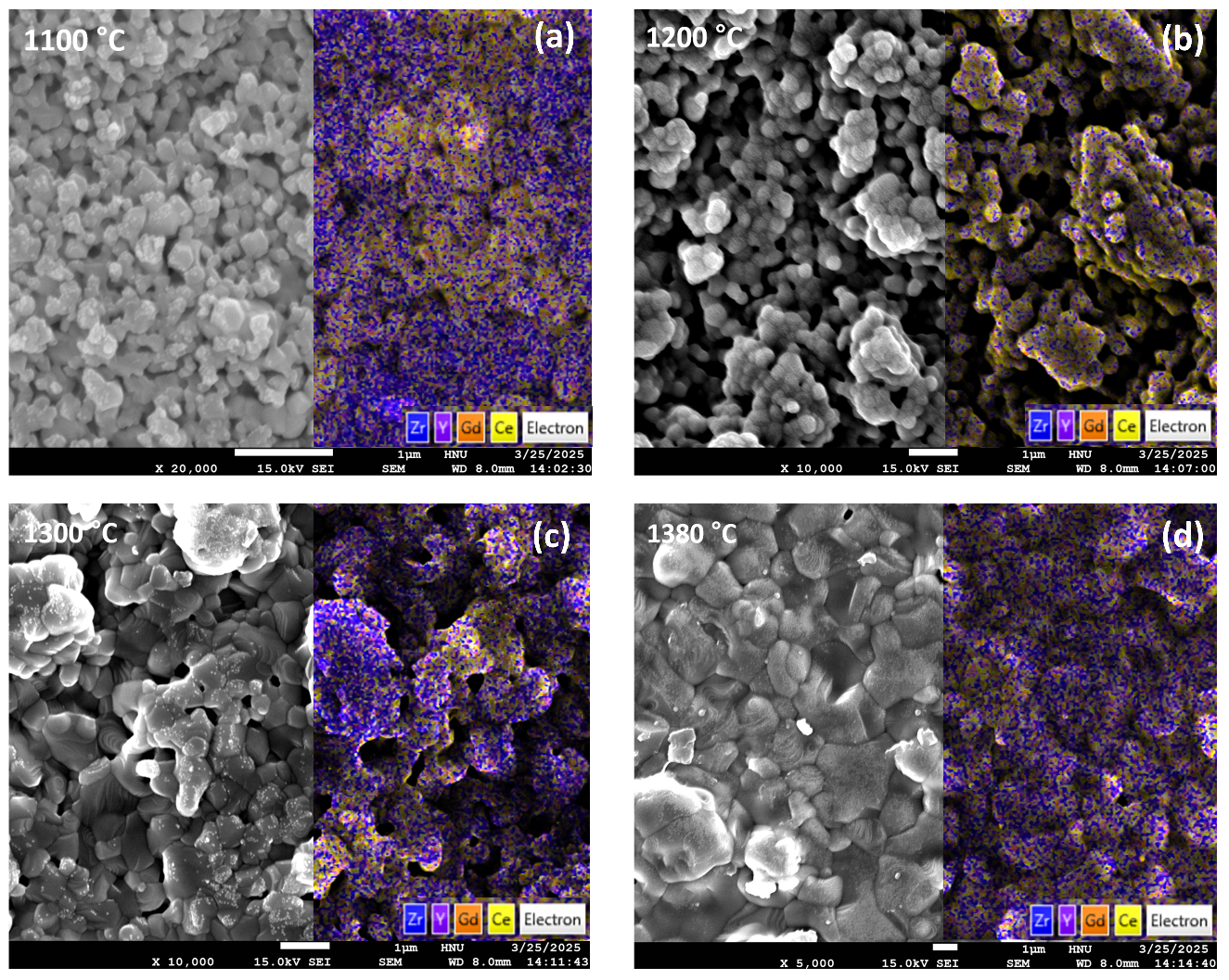


**Figure S10.** O 1s XPS spectra and calculated Oads/Olat ratios for composite intermediate layers with varying YSZ-to-GDC ratios.

**5YSZ5GDC**

**7YSZ3GDC**

**1YSZ9GDC**

**2YSZ8GDC**

**Table S3.** XPS O 1s Peak Fitting Parameters and Oxygen Species Distribution of YSZ-GDC Composite Electrolyte Layers

| Sample | Binding state | Binding energy[eV] | FWHM[eV] | Area | Oads/Olat |
| --- | --- | --- | --- | --- | --- |
| 7YSZ3GDC | Olat  Oads  Oho | 528.8 | 1.37 | 168253 | 0.32 |
|  |  | 531.75  530.35 | 1.65  1.36 | 53728  34428 |  |
| 5YSZ5GDC | Olat  Oads  Oho | 528.65 | 1.39 | 141452 | 0.35 |
|  |  | 531.5  530.2 | 1.57  1.33 | 50461  26568 |  |
| 2YSZ8GDC | Olat  Oads  Oho | 528.65 | 1.39 | 123208 | 0.53 |
|  |  | 531.75  530.25 | 1.83  1.48 | 65696  34279 |  |
| 7YSZ3GDC | Olat  Oads  Oho | 528.75 | 1.64 | 120938 | 0.57 |
|  |  | 531.75  530.35 | 1.77  1.32 | 68675  31819 |  |

**Table S4.** Calculated Oads/Olat ratios on GDC surface with and without 5YSZ5GDC composite intermediate layer.

| Sample | Binding state | Binding energy[eV] | FWHM[eV] | Area | Oads/Olat |
| --- | --- | --- | --- | --- | --- |
| **GDC**  (YSZ/GDC) | Olat  Oads  Oho | 528.5 | 1.5 | 86213 | 0.67 |
|  |  | 531.8  530 | 2.0  1.9 | 58513  54044 |  |
| **GDC**  (YSZ/5YSZ5GDC/GDC) | Olat  Oads  Oho | 528.4 | 1.2 | 42297 | 1.27 |
|  |  | 531.6  529.8 | 1.83  1.84 | 53843  39748 |  |

**Figure S11.** Raman spectra of the GDC surfaces for cells with and without the 5YSZ5GDC composite interlayer.


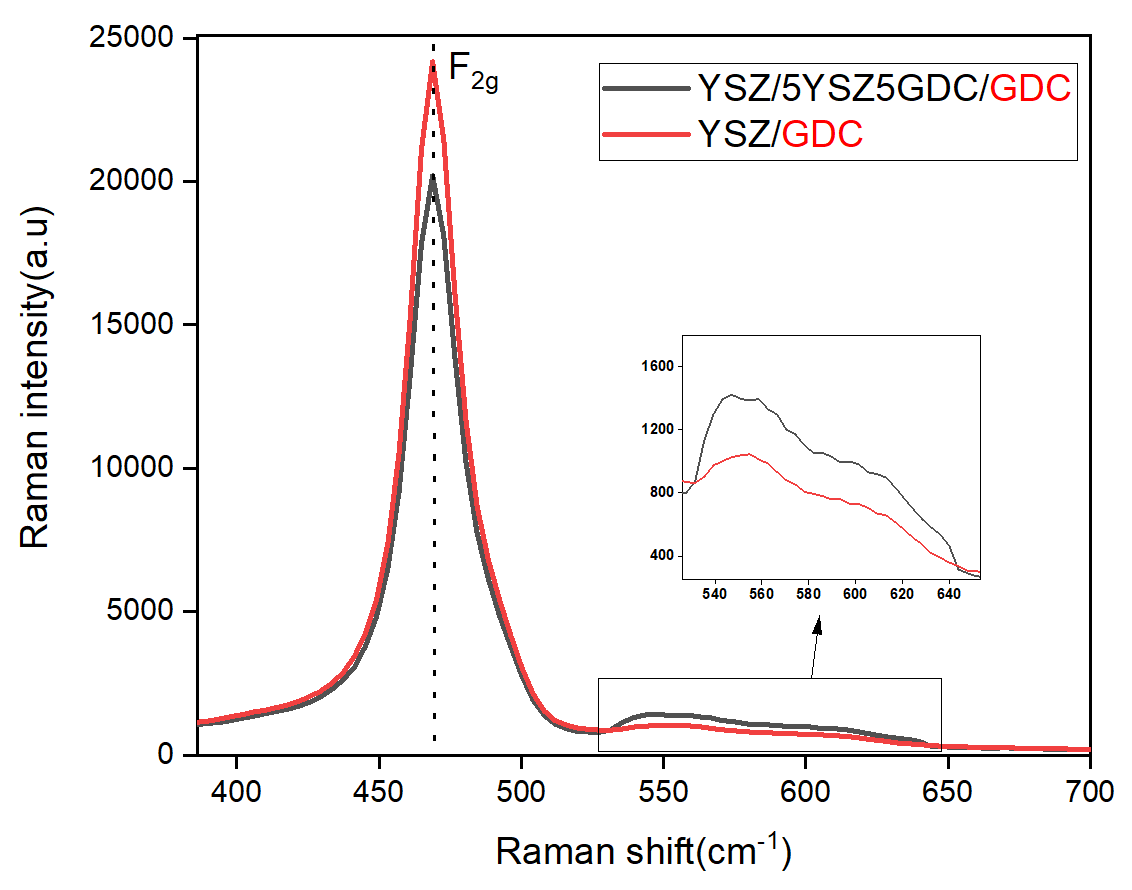


**Figure S12.** Surface images and work function analysis using KPFM and CPD (Contact Potential Difference) measurements: (a) Equation used for calculating work function, (b) GDC surface without composite intermediate layer, (c) GDC surface with 5YSZ5GDC composite intermediate layer.

1. Sample work function (Φsample) = Tip work function (Φtip,Tip used: Pt/Ir, 5.1eV) + eV_CPD_

Φsample is the work function of the sample under inspection, Φtip is the work function of the Kelvin probe tip, e is the elementary charge, and VCPD is the measured value of contact potential difference.


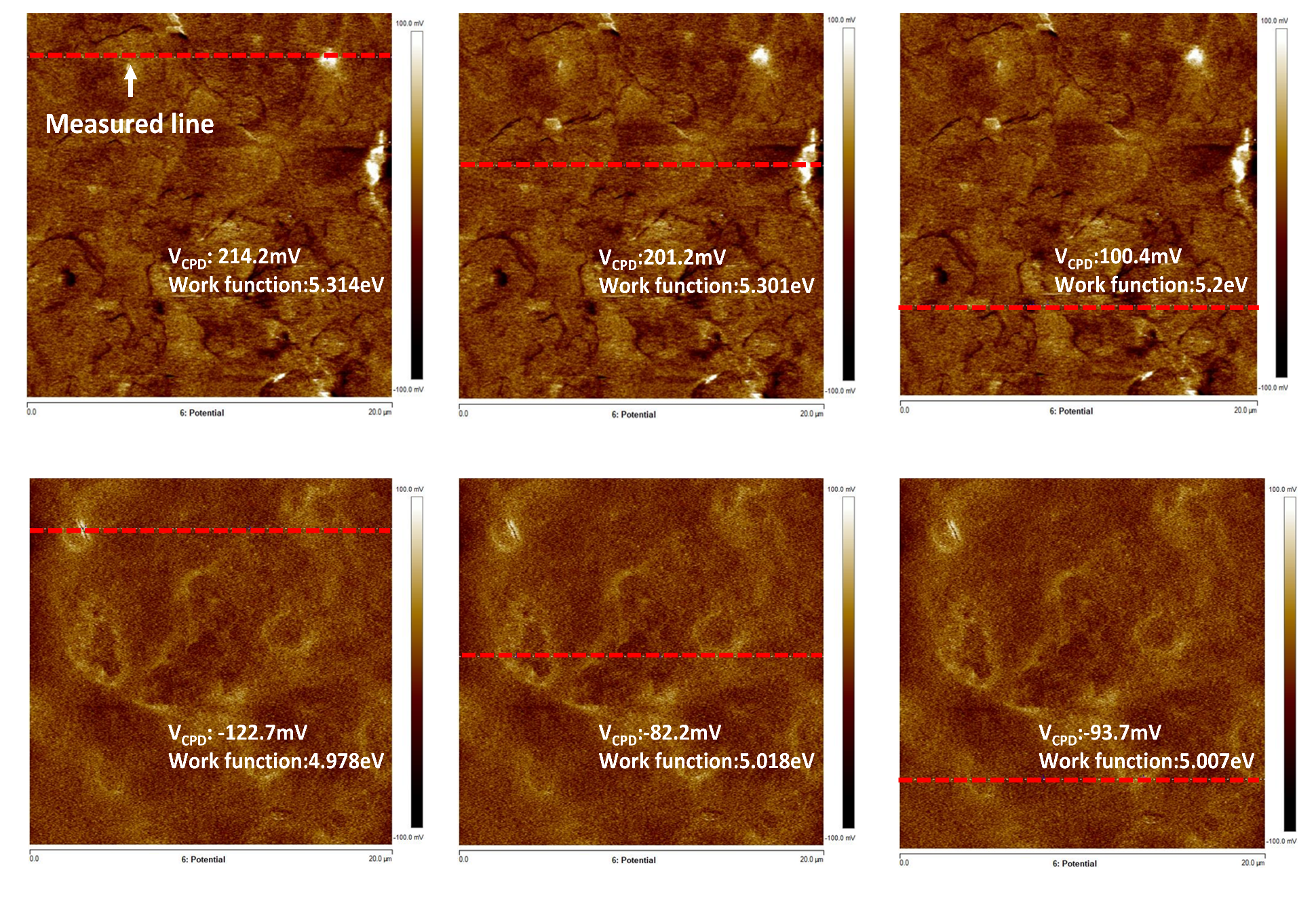
 (b)

(c)

**Figure S13.** Nyquist plots obtained for various composite electrolyte ratios at different temperatures.

**Table S5.** Detailed DRT Analysis of Electrochemical Processes

Table S5a) DRT process definitions with corresponding time constant ranges, frequency ranges, and physical assignments for electrochemical impedance analysis.

| Process | τ Range [s] | Frequency Range | Physical Assignment |
| --- | --- | --- | --- |
| P1 | 1.0×10⁻⁶ – 4.0×10⁻⁵ | 3.98 kHz – 159 kHz | O²⁻ transport across the electrode/electrolyte interface |
| P2 | 4.0×10⁻⁵ – 3.0×10⁻³ | 53.1 Hz – 3.98 kHz | O²⁻ ion transport within the electrode |
| P3 | 3.0×10⁻³ – 1.0×10⁻¹ | 1.6 Hz – 53.1 Hz | CO₂ adsorption and gas phase conversion |

Table S5b. Deconvoluted resistance values from DRT analysis of cells with different composite interlayers at 800 °C and 1.8 V. The characteristic frequency for each process is derived from the corresponding relaxation time (τ) range (P1: 10^−6^ ~ 4×10^−5^, P2: 4×10^−5^ ~ 3×10^−3^ s, P3: 3×10^−3^ ~ 10^−1^ s)

| **Sample** | **Total Rp (Ω·cm²)** | **Rp1 (Ω·cm²)** | **Rp2 (Ω·cm²)** | **Rp3 (Ω·cm²)** |
| --- | --- | --- | --- | --- |
| **No Interlayer** | 2.984 | 0.585 | 1.436 | 0.964 |
| **1YSZ9GDC** | 0.582 | 0.366 | 0.134 | 0.083 |
| **2YSZ8GDC** | 0.329 | 0.223 | 0.031 | 0.074 |
| **5YSZ5GDC** | 0.37 | 0.27 | 0.1 | 0.001 |
| **7YSZ3GDC** | 0.485 | 0.355 | 0.13 | 0 |

Table S5c. Deconvoluted resistance values from DRT analysis of 2YSZ8GDC cells at different applied voltages at 800 °C. The characteristic frequency for each process is derived from the corresponding relaxation time (τ) range (P1: 10^−6^ ~ 4×10^−5^, P2: 4×10^−5^ ~ 3×10^−3^ s, P3: 3×10^−3^ ~ 10^−1^ s)

| **Voltage** | **Total Rp (Ω·cm²)** | **Rp1 (Ω·cm²)** | **Rp2 (Ω·cm²)** | **Rp3 (Ω·cm²)** |
| --- | --- | --- | --- | --- |
| **OCV** | 2.024 | 0.6478 | 1.2398 | 0.1363 |
| **1.2V** | 0.6819 | 0.453 | 0.2289 | 0 |
| **1.4V** | 0.6237 | 0.422 | 0.2017 | 0 |
| **1.6V** | 0.5005 | 0.3646 | 0.1359 | 0 |
| **1.8V** | 0.3287 | 0.2234 | 0.031 | 0.0743 |

Table S5d. Deconvoluted resistance values from DRT analysis of 2YSZ8GDC cells at different operating temperatures at 1.8 V. The characteristic frequency for each process is derived from the corresponding relaxation time (τ) range (P1: 10^−6^ ~ 4×10^−5^, P2: 4×10^−5^ ~ 3×10^−3^ s, P3: 3×10^−3^ ~ 10^−1^ s)

| **Temperature** | **Total Rp (Ω·cm²)** | **Rp1 (Ω·cm²)** | **Rp2 (Ω·cm²)** | **Rp3 (Ω·cm²)** |
| --- | --- | --- | --- | --- |
| **800°C** | 0.3287 | 0.2234 | 0.031 | 0.0743 |
| **700°C** | 0.381 | 0.2697 | 0.0798 | 0.0315 |
| **600°C** | 1.4703 | 0.3748 | 0.7978 | 0.2976 |
